# Development of a habit-based intervention to support healthy eating and physical activity behaviours for pregnant women with overweight or obesity: Healthy Habits in Pregnancy and Beyond (HHIPBe)

**DOI:** 10.1186/s12884-024-06945-7

**Published:** 2024-11-16

**Authors:** Julia McClelland, Dunla Gallagher, Sarah E Moore, Caroline McGirr, Rebecca J Beeken, Helen Croker, Kelly-Ann Eastwood, Roisin F O’Neill, Jayne V Woodside, Laura McGowan, Michelle C McKinley

**Affiliations:** 1 Centre for Public Health, Institute for Global Food Security, Institute of Clinical Sciences, Queen’s University Belfast, Royal Victoria Hospital, Block A, Belfast, UK; 2 Leeds Institute of Health Sciences, School of Medicine, University of Leeds, Leeds, UK; 3 World Cancer Research Fund, London, UK; 4 St Michael’s Hospital, University Hospitals Bristol and Weston NHS Foundation Trust, Southwell Street, Bristol, UK

**Keywords:** Obesity, Overweight, Intervention development, Pregnancy, Health, Behaviour, Behavioural intervention, Diet, Activity, Personal and public involvement (PPI), Gestational weight gain

## Abstract

**Background:**

The number of women entering pregnancy with overweight or obesity is increasing. This can increase the risk for excessive gestational weight gain (GWG) which is associated with health complications for mother and baby. There are limited evidence-based interventions within antenatal care settings to encourage healthy eating and physical activity behaviours and support women with managing GWG.

**Methods:**

A previous habit-based intervention ‘Ten Top Tips for a Healthy Weight’ (10TT) was adapted and made suitable for pregnancy in line with the Medical Research Council’s (MRC) complex intervention development guidelines. It involved three key activities: (1) identifying the evidence base; (2) identifying appropriate theory; and, (3) modelling processes. A core element was integrating lived experience via personal and public involvement (PPI).

**Results:**

The original 10TTs were adapted with PPI in line with current advice on nutrition and physical activity in pregnancy. New intervention materials were devised, including a leaflet and a logbook and app for self-monitoring to be delivered alongside a brief 1:1 conversation. Behaviour change techniques (BCTs) included in the new materials were coded using a number of behavioural taxonomies. An E-learning resource was created to help standardise the approach to delivery of the intervention and avoid stigmatising conversations.

**Conclusion:**

Following MRC guidance for the development of complex interventions alongside significant PPI allowed for the adaption of 10TT habit-based weight management intervention into the ‘Healthy Habits in Pregnancy and Beyond’ (HHIPBe) intervention. The feasibility and acceptability of implementing this intervention in the antenatal setting will be explored in a feasibility randomised controlled trial.

**Trial Registration:**

This study was registered on Clinical Trials as ‘Healthy Habits in Pregnancy and Beyond (HHIPBe)’ ClinicalTrials.gov Identifier: NCT04336878. The study was registered on 07/04/2020.

**Supplementary Information:**

The online version contains supplementary material available at 10.1186/s12884-024-06945-7.

## Contributions to the literature

- There is a gap in services to support women entering pregnancy at a higher weight. Scalable behaviour change interventions that can be implemented within the current healthcare system are needed.
- An evidence-based weight management intervention based on the principles of habit-formation, used previously in primary care (10 Top Tips for a Healthy Weight), was successfully adapted for pregnant women with overweight and obesity (Healthy Habits in Pregnancy and Beyond [HHIPBe]).
- Lived experience was critical to the development and framing of the HHIPBe intervention given the stigma pregnant women with overweight and obesity face within healthcare settings.

## Background

Women who enter pregnancy with overweight or obesity (obesity typically defined as a body mass index (BMI) ≥ 30 kg/m^2^) are at an increased risk for excessive gestational weight gain (GWG), which is associated with health complications for the mother and baby [1]. Currently, the UK and ROI do not have specific guidance on recommendations for GWG. However, the Institute of Medicine (IOM) guidelines are used frequently as a benchmark for appropriate GWG, with research suggesting there are increased health risks for inadequate or excessive GWG [2, 3]. There is limited evidence-based support available to women in the United Kingdom (UK) and Republic of Ireland (ROI) regarding healthy eating and physical activity behaviours, which may aid management of weight gain during pregnancy. Recent systematic reviews have examined the effective intervention strategies/components of dietary interventions and/or physical activity interventions to prevent excessive GWG [4–13]. Although a number of interventions have been effective at limiting GWG or improving nutrition and physical activity behaviours during pregnancy [5, 12, 14–18], many have been intensive, requiring women to have regular contact with the researchers/healthcare professionals. This level of intensity limits the potential for scale-up and implementation within current healthcare systems. Intensive interventions can be burdensome for participants, often resulting in high dropout rates and are costly and time-consuming to set-up [7, 11]. Since approximately half of all pregnant women in the UK have overweight or obesity and the prevalence is increasing [19, 20], it is important to identify whether lower intensity interventions can effectively support health-promoting behaviour change during pregnancy, whilst at the same time being cost-effective and suitable for implementation within current healthcare services.

Systematic review evidence has shown efficacy of habit-based behaviour change interventions for participants with overweight and obesity (modest but statistically significant effects) and habit-formation approaches offer theoretical potential for longer-term behaviour change [21, 22]. Habit theory proposes that *context-dependent* repetitions of a planned behaviour in response to a specific cue/context can lead to the development of ‘automaticity’, which is less reliant on motivation and intentions [23–25] and is specific to ‘habitual behaviour’. This makes habit-formation approaches attractive for behaviour change, as, theoretically, enacting the healthy planned behaviour becomes easier over time due to the development of automaticity, via these context-dependent repetitions [23, 24]. Previous habit-based interventions have been delivered within UK healthcare settings, and are adaptable in terms of intensity of delivery, which has relevance for scalability of implementation. For example, a brief habit-based intervention, called the ‘Ten Top Tips for a Healthy Weight’ (10TT) [26, 27] was conducted in primary care to support adults with obesity to manage weight and to develop healthy habitual behaviours in relation to management of healthy eating and physical activity [26, 28]. This brief intervention was successful at promoting weight loss; participants in the 10TT group had mean weight loss of 1.68 kg (SD 3.21) after 3 months compared with 0.84 kg (SD 2.83) in the ‘usual care’ group [26] and it was deemed as cost-effective as usual care [29]. Other successful dietary and/or physical activity-based interventions using habit theory as a mechanism for behaviour change have been conducted in home-based settings and with other population groups, including helping parents of young children to form healthy feeding habits [27, 30–32].

Given the potential of the 10TT habit-based intervention to support health behaviour change when delivered as a brief intervention in a healthcare setting, coupled with the lack of evidence-based and scalable interventions suitable for implementation in the antenatal period on the island of Ireland, we obtained funding to adapt the 10TT intervention for use during pregnancy. This paper reports the adaptation of the original 10TT habit-based weight loss intervention into an intervention suitable for use with a pregnant population living with overweight and obesity. The intervention was designed to target healthy eating and physical activity behaviours to aid management of GWG, delivered via routine antenatal care in the UK and ROI.

## Methods

The UK Medical Research Council (MRC) guidance on the development of complex interventions provided the framework for the development of the intervention adaptation (undertaken in 2018) [33]. The MRC guidance on developing and evaluating complex interventions states ‘best practice is to develop interventions systematically, using the best available evidence and appropriate theory’ [33]. The intervention should then be tested using a pilot or feasibility study before carrying out definitive evaluations of the intervention [33]. This manuscript focuses on stage one of the MRC guidance, specifically identifying the evidence base; identifying and developing appropriate theory; and, modelling process and outcomes. The intervention was developed in line with the TIDieR framework [34] and the completed GUIDED checklist can be seen in Additional File 1 [35].

The adaptation of the 10TT intervention for a pregnant population included consideration of the relevance of *participant-facing materials* and the resources and training required for delivery of the brief intervention by *healthcare professionals.*

### Identifying the evidence base

The need for this intervention was identified given increasing rates of overweight and obesity in pregnancy and the potential short and long-term implications of excess weight in pregnancy for mothers and babies, alongside consideration of current service provision and what support might be helpful for women at this time.

### Identify and develop appropriate theory

Given the recent success of habit-based interventions for weight-management [21] and the previously noted efficacy of the 10TT habit-based intervention with adults living with obesity in primary care in the UK, an adaptation of this 10TT intervention for use with a pregnant population was undertaken, focusing on habit theory [26]. In the initial stages of habit-formation, it is likely that additional behaviour change techniques (BCTs) will be needed to support enacting any new, planned healthy behaviours, for example ‘goal-setting’, ‘self-monitoring’ and ‘prompts and cues’ [36–38].

*Participant materials*: A rapid review was undertaken to inform the adaptation of the 10TT materials to a pregnancy context regarding the theoretical basis and behaviour change content. Systematic reviews of GWG interventions and individual RCTs that were published since the most recent systematic review (September 2021) were examined, with the focus being on identifying BCTs used in successful behavioural interventions that included a nutritional component (see Additional File 2 for search strategy). BCTs in the original 10TT intervention materials have previously been coded [39] and whilst adapting the intervention, the operationalisation of the BCTs for a pregnant population was considered. The adapted intervention materials were coded using: the CALO-RE taxonomy; the BCT taxonomy (v1) 93-items; and, the Oxford Food and Activity Behaviours (OxFAB) taxonomy [36, 40, 41]. The BCT taxonomy (v1) and OxFAB taxonomy were used to code the adapted intervention to capture BCTs such as, “habit-formation” and “impulse management- awareness of motives”, that were not included in the 40-item CALO-RE taxonomy. Intervention materials were initially coded by JMcC and reviewed and coded by MMcK, LMcG and DG to reach consensus (team with expertise in public health, nutrition, psychology and behavioural science). Additional team members, HC (dietetics and behaviour change) and RB (psychology, habit-formation), addressed any uncertainties with the coding to reach final consensus.

### Modelling process and outcomes

*Participant materials*: The materials used in the original 10TT intervention were adapted to ensure appropriateness for pregnancy [26]. The adapted intervention was called ‘Healthy Habits in Pregnancy and Beyond (HHIPBe)’. Key clinical documents and dietary recommendations were accessed to ensure the materials were based on the latest guidance on nutrition and physical activity in pregnancy. This included documents such as the National Institute for Health and Care Excellence (NICE) guidelines, Royal College of Obstetricians and Gynaecologists (RCOG) guidelines, Health Service Executive (HSE) and National Health Service (NHS) guidelines, as well as recent systematic reviews (published within the past 10 years) and recent studies on specific issues as required. Based on this evidence, a scientific rationale was created for each of the HHIPBe top tips. This was conducted as an iterative process with core team members (MMcK, LMcG, DG, CMcG and JMcC) in collaboration with other study investigators (KAE, RB, HC) and PPI^*¥*^ collaborators. *(*^*¥*^*Note: In Northern Ireland*,* PPI stands for Personal and Public Involvement due to integrated Health and Social Care systems*,* whereas in the rest of the UK it is referred to as ‘Patient and Public Involvement’ also known as ‘Patient and Public Involvement and Engagement’).* Details on the process of PPI collaboration are given below. The 10TT participant materials that were adapted for the HHIPBe intervention were: a leaflet, logbook and (mobile) app. For the app, a 10TT app created by Bond University [42] was adapted, with permission, into a progressive web app supported across PC, iOS and Android on Chrome, Safari, Opera and Edge browsers.

Given the mode of delivery of the intervention, via a brief 1:1 conversation with a healthcare professional, a specific e-learning training resource was also developed as described below.

*Healthcare professional training to deliver the brief 1:1 conversation*: The aim of the E-learning training resource was to provide support to midwives in delivering the intervention 1:1 brief conversation as part of antenatal care and to ensure fidelity of delivery across providers. Published literature on raising the issue of weight in a sensitive and non-stigmatising way was consulted alongside effective models for brief interventions in a healthcare context, particularly in relation to pregnancy [43–46] .

An E-learning resource was developed by the trial team and reviewed by study co-investigators. The content was based on the original 10TT intervention training, principles of habit-formation theory and clinical guidance in NI and ROI. To ensure that the training was consistent with resources already accessible to midwives, the content of some existing E-learning programmes relevant to weight management in pregnancy was reviewed and considered [47].

To facilitate visual learning, role-plays of how the delivery of the 1:1 brief conversation might happen in real life were developed by the trial team and reviewed by study co-investigators. Video recordings of these scenarios were produced in conjunction with PPI collaborators and simulated patients who participate in medical education activities, who acted out the roles of midwife/patient and were paid for their time.

The final E-learning tool was designed in collaboration with the E-learning Team in the School of Medicine, Dentistry and Biomedical Sciences at Queen’s University Belfast. The graphic design company responsible for developing the HHIPBe intervention leaflet provided permission for the leaflet design scheme to be replicated on the E-learning package to provide consistency of branding.

### Personal^¥^ and public involvement (PPI) in intervention development

PPI from those with lived experience is vital in the development of complex behavioural interventions to ensure they are relevant and acceptable to the target population [48]. PPI should contribute to all stages of the research cycle [49]. The PPI in this intervention development process is described according to the GRIPP2 SF (Guidance for Reporting Involvement of Patients and the Public (2nd version)- Short Form) sub-headings [50].

*Aim of PPI (GRIPP2 SF Heading)* -The aim of the PPI was to collaboratively involve those with lived experience in the development of an intervention to support women with overweight or obesity with healthy eating and physical activity behaviours during pregnancy, to aid management of GWG.

*Methods for PPI (GRIPP2 SF Heading)* - In order to engage PPI collaborators, posters and flyers (see Additional File 3) were displayed in the waiting areas of antenatal wards in four hospitals in NI and ROI. Women could contact the research team via email or telephone if they were interested in becoming a PPI collaborator for the study. Alongside this, letters were sent to women who had previously taken part in a research study at the Centre for Public Health, Queen’s University Belfast, who had consented to be contacted about future research involvement. The PPI collaborators were reimbursed for their time and travel expenses according to NIHR guidance [51].

*What was involved for PPI Collaborators? (GRIPP2 SF Heading)* – PPI were involved and will continue to be involved with various aspects of the study, however for the purposes of this paper we focused on the PPI input into the development of the intervention materials. During the adaptation of the 10TT intervention, the PPI collaborators were involved in shaping the intervention design and content via discussions, email, and written feedback for the following elements of the intervention: the study name; the content of the leaflet; the design of the leaflet; the content and design of the logbook; and user testing the app. For example, with regard to the design and content of the leaflet, once the initial revisions had been made to incorporate the latest available scientific and clinical evidence, PPI collaborators engaged with the research team to provide input via their preferred method of communication (e.g. telephone call/email/written feedback). PPI collaborators received a working draft of the introduction, tips and frequently asked questions sections of the leaflet. This was presented in a table with four columns after each section where they were asked to indicate if they ‘like it’, ‘it’s ok’ or ‘don’t like it’ and then provide ‘comments’ to explain their ratings. There was also a textbox at the end of the document for any additional comments.

## Results

The results from each stage of the MRC guidance resulting in development of the ‘Healthy Habits in Pregnancy and Beyond’ or ‘HHIPBe’ intervention are outlined below.

### Identifying the evidence base

#### Need

Epidemiological evidence indicated an increased prevalence of overweight and obesity among women of reproductive age, with data from NI showing that more than one in two women enter pregnancy with excess weight [20]. By 2025, globally it is expected that 21% of women will have obesity and 9% will have a BMI ≥ 35 kg/m^2^ [52]. In the UK, around 50% of pregnant women have overweight or obesity at the start of pregnancy with approximately 5% having a BMI over 35 kg/m^2^, a risk factor for maternal morbidity and mortality [53, 54]. However, entering pregnancy with a BMI ≥ 25 kg/m^2^ (excess weight) is also associated with a number of health risks for the mother and baby based on systematic review evidence [55–58]. Furthermore, maternal obesity (BMI ≥ 30 kg/m^2^) can increase the risk of developing a range of health complications throughout pregnancy for both the mother and the fetus including, gestational diabetes mellitus, gestational hypertension, pre-eclampsis, venous thromboembolism, labour complications, caesarian section, depression and premature birth [59]. Such evidence highlights the need to provide evidence-based services to support this pregnant population (BMI > 25 kg/m^2^) with health-promoting behaviours that may aid behavioural management of GWG and minimise risks. Yet, despite this, there is a lack of support available in NI and ROI regarding healthy eating and physical activity behaviours in pregnancy. Specifically, intervention approaches are needed that are evidence-based, theoretically strong and scalable, with regard to implementation in the current healthcare systems [24].

Evidence reviews on obesity and pregnancy point to the need for person-centred care, particularly for women with higher BMIs who regularly report negative experiences during antenatal care based on their weight [60]. This stigmatisation of women due to body shape or size was found to be highly prevalent across the reproductive stages (i.e., preconception, pregnancy and postpartum) in one review [61]. Therefore, despite pregnancy being considered a ‘window of opportunity’ because it is a time when women are in frequent contact with healthcare professionals and may be more receptive to health improvement messages, care is required to ensure interventions are supportive, and non-stigmatising [61–63]. Indeed, women with obesity have expressed a desire for additional behavioural support during their pregnancy yet, simultaneously, reported feeling stigmatised by healthcare professionals because of their weight, leading to a reluctance or hesitation in seeking support or advice on health or weight during pregnancy [64–66]. One way to ensure that any intervention is appropriately designed to meet the needs of the target population is to embed PPI within its development in order to ensure it is appropriate, sensitively and respectfully framed (described fully under Results: Modelling process and outcomes below).

### Identify and develop appropriate theory

#### Habit theory

Healthy diet and activity ‘habits’, i.e. habitual behaviours, are the goal of most weight-management programmes, but few draw explicitly on habit-formation theory. The essential feature of habits, once acquired, is that they are ‘automatic’ (i.e. require minimal willpower or deliberate effort) [67]. Research shows that repetition of a behaviour in a consistent context enables it to acquire automaticity, and once automaticity is established, it is more resistant to extinction than deliberative (intentional) behaviours [68, 69]. Habits are defined as ‘psychological dispositions to repeat past behaviour’ [70] and habit indicates “a process whereby exposure to a cue automatically triggers a non-conscious impulse to act due to the activation of a learned association between the cue and the action” [25, 71]. The main components of habit-formation include behavioural repetition, associated context cues and rewards [72]. According to Gardner et al., habit-formation involves three phases; the ‘initiation’ phase, ‘learning’ phase and the ‘stability’ phase [24, 67]. The ‘initiation’ phase involves planning what the intended behaviour is and which context/cue will be used to do the behaviour, the ‘learning’ phase involves repeating the behaviour in the selected context/cue consistently and, finally, the ‘stability’ phase is when the habit has been formed and the behaviour is automatic or ‘second nature’ to the person [24]. The habits formed may be more resistant to lapses and, therefore, are expected to be maintained over the long-term [23, 71–73].

The first habit-based behavioural intervention promoting a set of negative energy-balance behaviours via a leaflet, alongside advice on repetition, context-stability and self-monitoring showed significantly greater weight loss in the habit group compared to the control condition (habit group: -2.0 kg; control: -0.4 kg) at 8 weeks follow-up; and, unusually, for behavioural treatments, modest weight loss continued after the end of the active treatment period, up to 32 weeks [27]. Weight loss was associated with increased ‘automaticity’ of behaviours, suggesting that habit-formation underpinned the effectiveness of the intervention [27]. These findings were confirmed in a definitive RCT in adults with obesity drawn from primary care (‘Ten Top Tips’ [10TT] RCT), where 10TT was explained by healthcare staff (approx. 25 minutes), with no further clinical contact [26]. The 10TT group lost significantly more weight over a three month intervention period compared to those receiving ‘usual care’ and, at 24 months, the 10TT group maintained their weight loss and showed significant gains in automaticity; making this an effective, low intensity and low-cost treatment option (£23 per participant) [26, 28].

Habit-formation theory is a relatively novel theory to underpin lifestyle interventions for weight management [23, 71, 72]. It has been used successfully in weight management interventions for various population groups [21], but has not been tested via behavioural intervention in a pregnant population. Alongside this focus on the theoretical basis for the behavioural intervention, existing evidence regarding the BCTs associated with intervention effectiveness in previous GWG studies was examined, given additional BCTs are likely to aid enactment of new, planned, healthy behaviours in the initial stages of habit-formation [36, 37, 39].

#### BCTs

A rapid review was conducted during the intervention adaptation to explore the components of successful GWG interventions. Of the 22 interventions that reported BCT content, the most commonly used BCTs were goal setting, self-monitoring, feedback on behaviour and barrier identification/problem-solving. Habit-formation has also been identified as an important BCT in weight management interventions [21]. Although “habit-formation” is described as a BCT in the BCT Taxonomy (v1): 93 hierarchically-clustered techniques, there are additional BCTs which may support the movement through the different phases of habit-formation [36, 71]. Gardner and Rebar (2019) examined the BCTs which were commonly used to promote habit-formation in previous habit-based interventions and found the following BCTs were used most frequently: “habit-formation”, “use prompts and cues, “action planning”, “provide instruction on how to perform the behaviour”, “set behavioural goals” and “self-monitor behaviour” [36, 71]. Aside from reporting frequency of use, there was limited research linking BCT usage with effectiveness of the interventions. Two systematic reviews examined the most effective BCTs used in weight management in pregnancy interventions [37, 74]. Soltani et al. (2016) found the following BCT categories to be the most commonly used in interventions that were successful at limiting GWG; “feedback and monitoring”, “shaping knowledge”, “goals and planning”, “repetition and substitution”, “antecedents” and “comparison of behaviours” [37]. For interventions which focused on diet or diet *and* physical activity, the most successful BCT categories were “feedback and monitoring”, “shaping knowledge”, and “goals and planning” [37]. This is important as planning and self-monitoring are key elements of habit-formation theory [23]. In addition, an earlier systematic review by Hill et al. (2013) which used the CALO-RE taxonomy to code the BCTs embedded in pregnancy weight management interventions, found “providing information on the consequences of behaviour to the individual”, “provide rewards contingent on successful behaviour”, “prompt self-monitoring of behaviour” and “motivational interviewing” were the most effective BCTs used to help limit excessive GWG [40, 74]. Another systematic review which focused on exclusively digital health interventions in pregnancy, found that interventions which included a greater number of BCTs (particularly involving goal setting and self-monitoring) were more effective than others [75].

Of the original 10TT BCTs coded by Kliemann et al., all were retained in the adapted intervention (HHIPBe) aside from ‘goal setting (outcome)’ and ‘‘prompt review of outcome goals’ (see Table 1 for full details on BCTs in 10TT and HHIPBe) [39]. This was because the HHIPBe intervention focuses on turning the ten tips into habitual behaviours, rather than focusing on weight as an outcome goal, therefore women were only encouraged to set goals in relation to their behaviour. The ‘prompt self-monitoring of behavioural outcome’ BCT was retained as participants were given the option to record their weight throughout their pregnancy, if they wished. BCTs were integrated throughout the intervention materials, for example, “teach to use prompts/cues” was incorporated in the HHIPBe leaflet – “Add reminders to your environment and be prepared to carry out your habit (e.g. place this leaflet in a prominent position and have fruit available each day at breakfast).”

**Table 1:** Summary of the BCTs used in the original 10TT intervention and the adapted HHIPBe intervention

10TT Intervention^a^	CALO-RE Taxonomy	HHIPBe Intervention materials	CALO-RE Taxonomy	Additional BCTs coded BCT Taxonomy (v1)	Additional BCTs (OxFAB)^b^
*10TT Leaflet*	1 Provide information on consequences of behaviour in general 5 Goal setting (behaviour) 6 Goal setting (outcome) 7 Action planning 9 Set graded tasks 10 Prompt review of behavioural goals 11 Prompt review of outcome goals 16 Prompt self-monitoring of behaviour 17 Prompt self-monitoring of behavioural outcome 20 Provide information on where and when to perform the behaviour 23 Teach to use prompts/cues 24 Environmental restructuring 26 Prompt practice 21 Provide instruction on how to perform the behaviour	*HHIPBe Leaflet*	1 Provide information on consequences of behaviour in general 2 Provide information on consequences of behaviour to the individual 5 Goal setting (behaviour) 7 Action planning 8 Barrier identification/problem-solving 9 Set graded tasks 10 Prompt review of behavioural goals 16 Prompt self-monitoring of behaviour 17 Prompt self-monitoring of behavioural outcome 19 Provide feedback on performance 20 Provide information on where and when to perform the behaviour 21 Provide instruction on how to perform the behaviour 23 Teach to use prompts/cues 24 Environmental restructuring 26 Prompt practice	7.1 Prompts and cues 8.2 Behaviour substitution 8.3 Habit-formation 8.4 Habit reversal 9.1 Credible source 11.3 Conserving mental resources 12.4 Distraction	- Impulse management: awareness of motives - Information seeking - Planning content
*10TT Logbook*	7 Action planning 9 Set graded tasks 10 Prompt review of behavioural goals 11 Prompt review of outcome goals 16 Prompt self-monitoring of behaviour 17 Prompt self-monitoring of behavioural outcome 22 Model/Demonstrate the behaviour 23 Teach to use prompts/cues 26 Prompt practice	*HHIPBe Logbook*	5 Goal setting (behaviour) 7 Action planning 8 Barrier identification/problem-solving 10 Prompt review of behavioural goals 16 Prompt self-monitoring of behaviour 17 Prompt self-monitoring of behavioural outcome 21 Provide instruction on how to perform the behaviour 26 Prompt practice	8.3 Habit-formation	
*Not used in 10TT RCT*	-	*HHIPBe* *App*	1 Provide information on consequences of behaviour in general 2 Provide information on consequences of behaviour to the individual 5 Goal setting (behaviour) 7 Action planning 8 Barrier identification/problem-solving 9 Set graded tasks 10 Prompt review of behavioural goals 16 Prompt self-monitoring of behaviour 17 Prompt self-monitoring of behavioural outcome 19 Provide feedback on performance 20 Provide information on where and when to perform the behaviour 21 Provide instruction on how to perform the behaviour 23 Teach to use prompts/cues 24 Environmental restructuring 26 Prompt practice	2.7 Feedback on outcome of behaviour 6.1 Demonstration of the behaviour 8.2 Behaviour substitution 8.3 Habit-formation 8.4 Habit reversal 9.1 Credible source 11.3 Conserving mental resources 12.4 Distraction	- Impulse management: awareness of motives - Information seeking - Planning content
*Not coded in 10TT RCT*	*-*	*Delivery of Intervention by Healthcare professional (Midwife or Researcher)*^c^	1 Provide information on consequences of behaviour in general 2 Provide information on consequences of behaviour to the individual 5 Goal setting (behaviour) 7 Action planning 8 Barrier identification/problem-solving 9 Set graded tasks 10 Prompt review of behavioural goals 16 Prompt self-monitoring of behaviour 17 Prompt self-monitoring of behavioural outcome 20 Provide information on where and when to perform the behaviour 21 Provide instruction on how to perform the behaviour 22 Model/Demonstrate the behaviour 23 Teach to use prompts/cues 24 Environmental restructuring 26 Prompt practice 28 Plan social support/social change	8.2 Behaviour substitution 8.3 Habit-formation 8.4 Habit reversal 9.1 Credible source 12.4 Distraction	- Impulse management: awareness of motives - Information seeking

^a^BCTs reported from (Kliemann, 2017) [39]

^b^OxFab taxonomy: behavioural strategies used by individuals rather than structured intervention techniques in weight control interventions

^c^The BCTs incorporated in the intervention delivery vary depending on which tips were discussed in detail

### Modelling process and outcomes

This section details how the 10TT intervention content was adapted for pregnancy and illustrates the role of PPI collaborators from the target population in shaping intervention design and content.

*PPI Results/Outcomes (GRIPP2 SF Heading)* - Women who came forward via the posters tended to either not have experience of living at a higher weight (i.e. a BMI ≥ 25 kg/m^2^) during a recent pregnancy, or wanted to take *part in the research* rather than collaborate in *developing the intervention*. Seven PPI collaborators were recruited from letters sent to previous research participants, two were recruited via word of mouth and one via the posters. Of these, ten initially engaged, and six became actively involved as PPI collaborators throughout. The PPI collaborators chose the name for the study – ‘Healthy Habits in Pregnancy and Beyond’ or ‘HHIPBe’. PPI collaborators also significantly influenced the content and design of the HHIPBe intervention materials.

#### Development of the HHIPBe participant leaflet

**Fig. 1:**
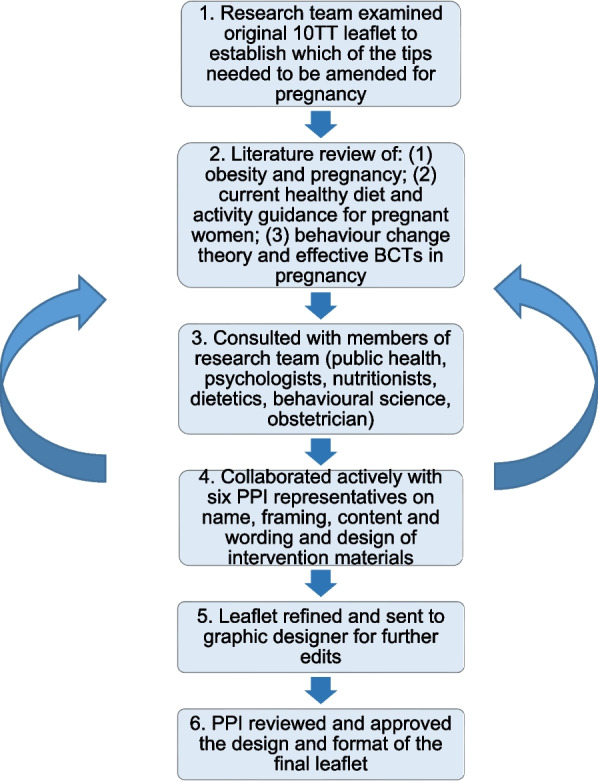
Summary of the development of the adapted HHIPBe participant leaflet

*Participant materials*: Figure 1 outlines the process of adapting the original 10TT intervention leaflet using an iterative process of review and amendment by the research team (MMcK, LMcG, DG, CMcG and JMcC) and PPI collaborators.

Following initial review of the original 10TT leaflet by the study team a draft adaptation of the leaflet was initially created by the research team. The adapted leaflet provided a scientific rationale for each of the top tips modified in line with pregnancy-specific available guidance (see Table 2 for two examples (out of ten)).

**Table 2:** Example of how the tips were adapted from the original 10TT to be appropriate for pregnancy

10TT original tip name	Adapted for pregnancy	Detail and adaptions for pregnancy and scientific rationale
**Walk off the weight**	**Get walking**	The name of this tip has been changed to avoid encouraging weight loss. There is no specific guidance of 10,000 steps recommended for pregnant women. Some of the handy hints have been changed to avoid the focus on calories. The recommendations for physical activity are the same in Ireland as in the UK [76, 77]. Physical activity is important throughout life. The recommendations are 150 min of moderate physical activity a week [77]. NICE guidelines recommend that pregnant women who have a BMI over 30 kg/m^2^ should have a “brisk walk or moderate activity for at least 30 minutes on at least 5 days” [78, 79]. The hints for this tip are in line with the ‘weight management before, during and after pregnancy’ NICE Guidance [78]. Being active during pregnancy helps to control GWG, reduces hypertensive disorders and reduces risk of gestational diabetes as well as improving cardiovascular fitness, sleep and mood [80]. Being active can also help women to cope and be physically fit for labour [81].
**Think about your drinks**	**Think about your drinks**	This content of this tip has been rewritten to make it appropriate for pregnancy. It is recommended to drink 6–8 glasses of fluid a day to stay hydrated before and after pregnancy as it can help with nausea [82–84]. Caffeine should be limited in pregnancy to 200 mg a day as it can disrupt the uptake of nutrients and it has been associated with a low birth weight [83, 85, 86]. Drinking alcohol in pregnancy has been associated with a number of adverse health effects and the safest approach is to abstain from alcohol [85, 87, 88]. Excessive alcohol consumption is linked with foetal alcohol syndrome [85, 89, 90].

The main consideration when adapting the 10TT leaflet was to remove the focus on weight loss, as this is not appropriate for a pregnancy intervention [78]. Many of the ‘handy hints’ for the tips included calorie information which was removed. Practical examples in the tips were revised to be specific for pregnancy, for example, the original leaflet suggested standing on a bus while travelling for the “up on your feet” tip, however, this would not be advisable for pregnant women for safety reasons. The leaflet was also modified to include relevant food safety advice such as guidance on caffeine intake during pregnancy.

**Fig. 2:**
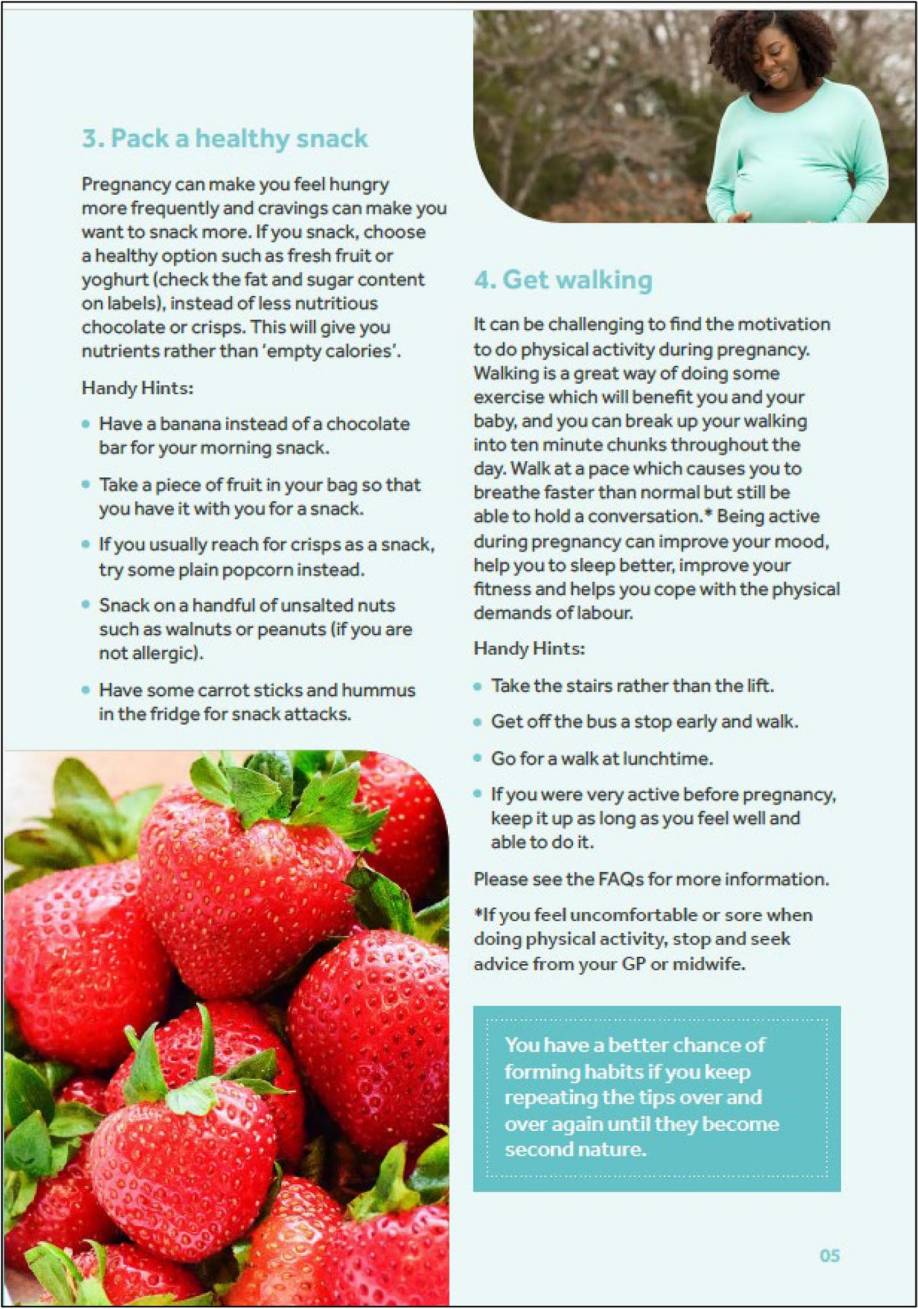
Example page from the adapted HHIPBe participant leaflet

Figure 2 presents an example page from the final version of the leaflet which was designed and printed by a graphic design company. The PPI collaborators were generally positive about the first draft of the participant leaflet; they found it was clear and concise with a good balance of encouragement and information. PPI collaborators created the frequently asked questions section of the leaflet and helped identify pregnancy specific challenges such as cravings, nausea and heartburn, with examples added on how to deal with these.

PPI collaborators also provided guidance on the terminology to describe weight throughout the intervention for example, using person-first language such as “having excess weight” rather than “being obese”. PPI collaborators suggested making the language more welcoming and empathetic, particularly in the introduction section of the leaflet, and wanted more practical suggestions to help facilitate the 10 tips becoming ‘habitual’. They also suggested including more examples for pregnant women who are working/in employment, as well as for women who do not work and, whilst they liked the design and use of images in the leaflet, they recommended a change of font colour to improve readability. The final leaflet consisted of 12 pages and included introductory text and information on why it is important to manage weight during pregnancy, ten tips with a description of each of the tips and some ‘handy hints’ on how to achieve the tips, a frequently asked questions section, a shopping guide, links to further information and an example tick sheet. The leaflet encourages participants to use the logbook or app to log their progress with the tips.

#### Development of the HHIPBe Participant Logbook

Similar to the leaflet, the HHIPBe logbook was adapted from the original 10TT logbook and reviewed/redesigned by members of the research team and PPI collaborators. The original 10TT intervention included a logbook for participants to monitor their daily progress with the ten tips and provided a space to plan how they would achieve each of the tips. The final logbook was designed and printed by a graphic design company in the same style as the HHIPBe leaflet.

In line with the leaflet, PPI collaborators modifications to the HHIPBe logbook included ensuring empathetic and friendly language was used, such as, “*we hope you find it useful to use alongside the HHIPBe leaflet*” and “*developing habits may require some effort at first*,* but if you follow the tips every day*,* and do them in the same place or at the same time*,* they will become automatic and easier to stick to*”.

**Fig. 3:**
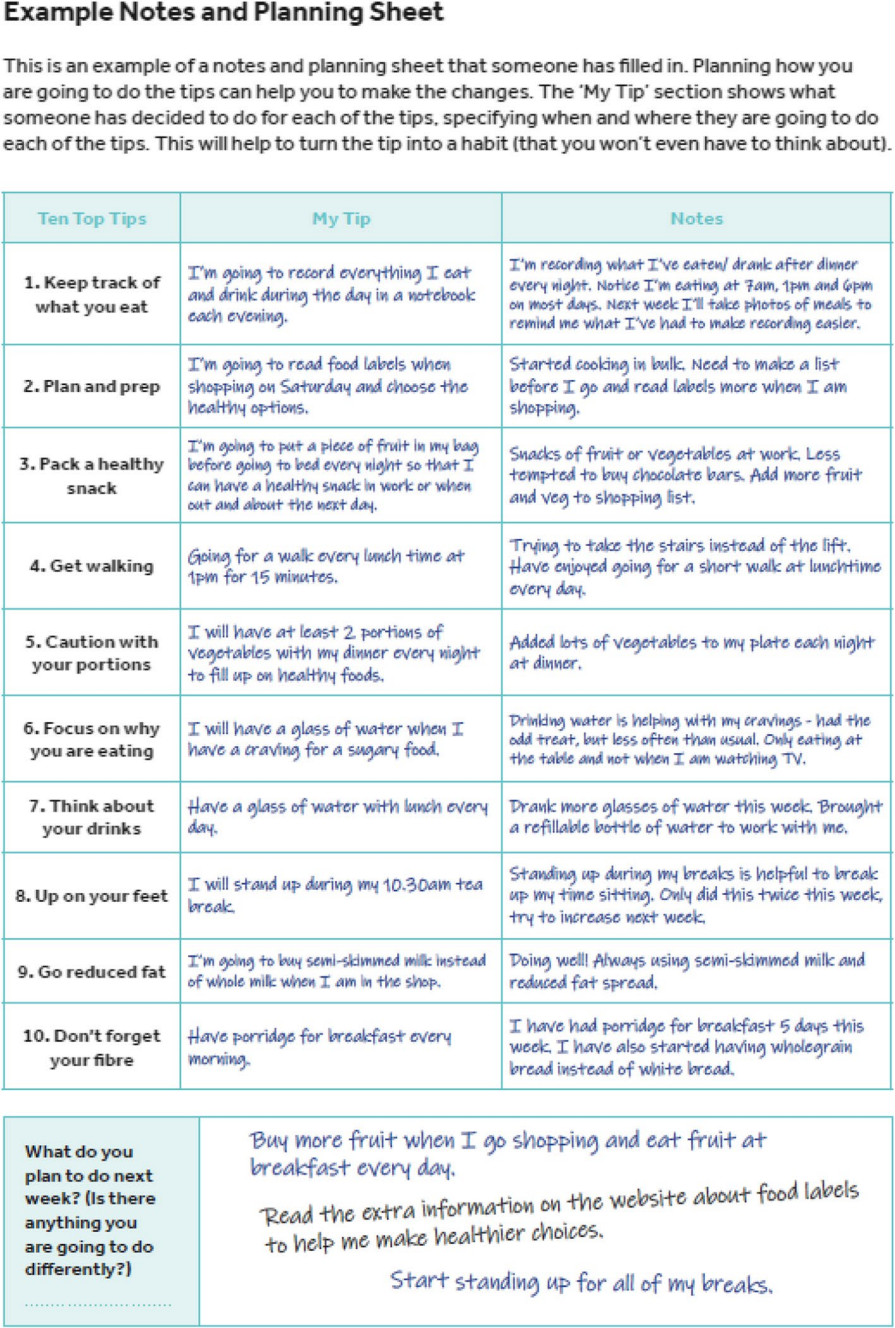
Example notes and planning sheet from the HHIPBe participant logbook

**Fig. 4:**
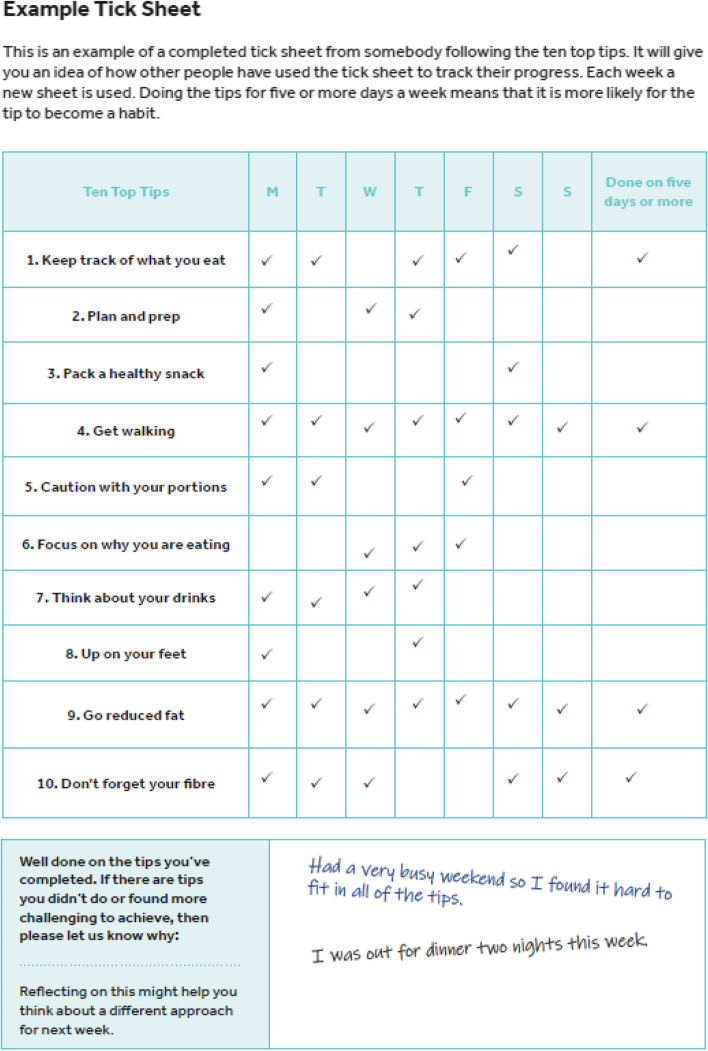
Example tick sheet from the HHIPBe participant logbook

An additional column was added to the notes and planning sheets, entitled “my tip”, which allowed participants to make the tip specific to them and set specific goals in relation to the tip. Alternative examples were incorporated to ensure they were suitable for pregnancy and an additional section was added to the tick sheets to allow participants to reflect on the past week, and make any notes on the tips that they were struggling with. Figures 3 and 4 show examples of the notes and planning sheet and tick sheet from the HHIPBe participant logbook.

The ‘record your weight’ section was also amended; in the original 10TT logbook, participants were encouraged to record their weight daily and it was included in the tick sheets. For the HHIPBe logbook, this section was repositioned to the back of the logbook and was optional for women. Women were encouraged to record their weight no more frequently than weekly and to speak to a healthcare professional if they noticed any weight loss or had any concerns about their weight. One PPI collaborator suggested expanding the explanation on the ‘recording your weight’ section and why it was needed, which was subsequently added into the logbook. Information on adequate GWG according to the IOM guidelines was also added to the leaflet [3].

PPI collaborators found the example notes, planning sheet and tick sheet helpful and liked that the example sheet was ‘realistic’, as it showed that someone did not achieve all of the tips. They liked the format of the logbook, however, wanted a larger space to write in goals and notes. The size of the logbook was changed from A5 to A4 based on PPI feedback to ensure there was enough space for the participants to make written notes and set goals in the logbook. All PPI collaborators comments were incorporated before the final logbook was printed.

#### Development of the HHIPBe participant (mobile) app

The app aimed to be user-friendly, have the same functionality as the logbook and offer an alternative to using the hard copy (A4) logbook and/or leaflet, given apps offer a convenient ‘anyplace, anytime’ way of accessing advice or monitoring tools [91–93]. All of the information included in the leaflet is included in the app but it also allows participants to log their progress with the tips. Participants can choose to use the app instead of the logbook.

The research team reviewed the list of essential features on the 10TT app, and modified it in-line with the HHIPBe versions of the leaflet and logbook [94]. Three PPI collaborators tested the HHIPBe app, helping to resolve initial issues with registering and logging onto the app. PPI collaborators felt the app would be useful, liked the app functionality and layout and suggested minor wording and layout edits. A tutorial was co-created with instructions on how to use the app based on input from PPI collaborators.

The app contained a small number of additional features compared with the paper logbook. Firstly, it had a graphical function to allow the participants to observe their weight gain (in Kg or stones and pounds) trajectory throughout pregnancy, the weight tracking function could be turned off. Secondly, the original 10TT app sent daily notifications to the participants, which resulted in mixed feedback with some participants finding the frequency ‘*annoying*’ [94]. Therefore, weekly email notifications were scheduled for HHIPBe participants (who registered an account with the app) to encourage the women to track their progress and encourage engagement. The weekly notification sent to the participants read:

*“Have you recorded your progress towards achieving the Ten Top Tips? You can do this by logging into the Healthy Habits in Pregnancy and Beyond app. Keeping a note of your progress will increase your success in developing healthy habits. Don’t worry if you forgot to log anything last week*,* it’s easy to add to past days. Why don’t you start now?”.*

These additional features expanded the BCTs included in the logbook with regard to increased feedback on behaviour and additional prompting to monitor behaviour or outcomes of behaviour, as summarised in Table 1.

#### Healthcare professional training to deliver the HHIPBe brief 1:1 conversation

The overall HHIPBe intervention consisted of a one-off, brief (15–20 min) 1:1 conversation with a trained professional at the outset. During this 1:1 session, the trained professional explains the process of habit formation, encourages the woman to consider what habits they might focus on initially and goes through the leaflet, logbook and app with them. Women continue with their routine antenatal appointments as usual and are encouraged to get in touch with the antenatal care team if they have any questions or concerns at any time. The original 10TT intervention was delivered by a trained nurse or healthcare assistant during a dedicated face-to-face 20–25 min (approx.) session in a primary care setting [26]. Based on discussions with midwives in the early stages of intervention adaptation, it was desirable to reduce the length of the 1:1 conversation to take account of service pressures and increase the likelihood that the intervention could be incorporated into routine antenatal care delivery.

Previous research has found that midwives find it difficult to discuss weight with women [95]; a perceived lack of adequate and available resources, equipment and training to address the psychological and physiological needs of mothers with a high BMI [96] and concerns over how to communicate about the issue without impacting the patient/caregiver relationship and ‘normalise’ obesity are prevalent [95]. This aligns with the views of women with a high BMI, who also report a fine balance between healthcare practitioners outlining the risks and management of obesity in pregnancy, and the psychological and potential stigmatising impact of this [97]. Guidelines have been developed around the use of language to address weight in pregnancy [95–97] in order to avoid stigmatisation of individuals [45]. These guidelines highlight the 5 As framework (ask, assess, advise, agree, assist) to guide conversations between healthcare practitioners and patients and facilitate the discussion of GWG in a non-judgemental manner. Recent evidence illustrates this tool can be effective at initiating these types of conversations with patients [45, 98, 99] and so it was selected to form the basis of the HHIPBe training.

The content of several E-learning programmes that were already accessible to midwives was considered when defining the content of the training. These included resources developed by the Canadian Obesity Network on the 5As framework for obesity weight management [99] and the modified 5As framework for healthy pregnancy weight gain [45]. We also reviewed the ‘Making every contact count’ training [47] available to healthcare professionals in ROI and a similar E-learning tool developed by the Public Health Agency NI [100]^,^ both of which were designed to help healthcare professionals undertake brief interventions to promote healthy behavioural change.

Overall, the healthcare professional training covered: background information on why managing weight during pregnancy is important; the principles of habit-formation; the intervention materials and how to introduce them to participants; and, how to set SMART (Specific, Measurable, Achievable, Realistic, Timely) goals to aid the establishment of new health-promoting behaviours. The training included a section on how to discuss weight sensitively with women. It included role-plays for different scenarios the intervention facilitator might face when delivering the intervention and demonstrated key aspects of delivery including: asking an open question; showing empathy; active listening and reflecting when discussing the patient’s previous weight management experiences; problem solving; discussing motivation for behaviour change; talking through a few HHIPBe tips; and setting a goal. A checklist for the 1:1 conversation facilitator and notecards to guide the key elements of the conversation were developed to support fidelity of delivery. The brief 1:1 conversation reinforced the BCTs in the participant intervention materials (leaflet, logbook, app) and provided additional BCTs, for example, engaging in effective goal-setting, problem solving (encouraging the participant to think of potential barriers and enablers to repeating the chosen behaviour in a consistent context to facilitate habit-formation), and action planning.

The E-learning package took 1–2 h to complete and could be completed over multiple sessions. Intermittent questions to test understanding of the content were developed by the trial team and included in the resource. A certificate was provided at the end.

**Fig. 5:**
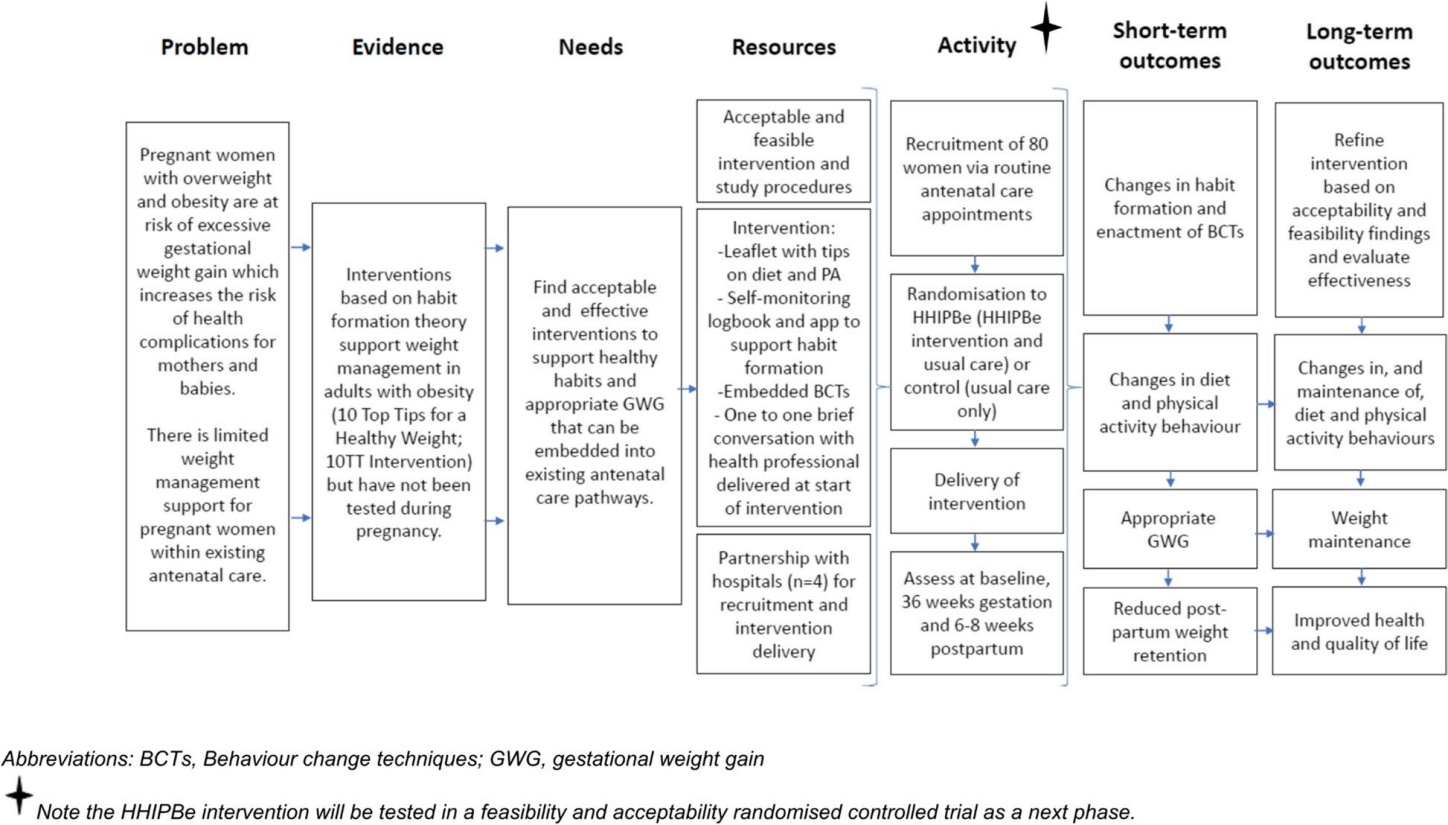
HHIPBe Logic Model

A logic model provided an overview of how the HHIPBe intervention was intended to facilitate positive health behaviour change (diet and physical activity) in pregnant women with overweight and/or obesity (Fig. 5).

## Discussion

This paper describes the adaptation of the ‘Ten Top Tips for a Healthy Weight’ (10TT) brief, habit-based weight loss intervention which was originally tested in a primary care setting with adults with obesity, into the ‘Healthy Habits in Pregnancy and Beyond’ (HHIPBe) intervention for pregnant women with overweight or obesity.

The adaptation took approximately 12 months. A significant strength to this research was the PPI in all stages of intervention adaption and development. This was alongside expert consultation, and reviewing of the evidence base underpinning pregnancy-specific guidelines for nutrition and physical activity behaviours in pregnancy and clinical guidance, with habit-formation theory guiding the theoretical basis for achieving behaviour change [101]. This resulted in the creation of a habit-based intervention for pregnant women with overweight and/or obesity, to support a range of 10 healthy eating and physical activity behaviours, to aid management of GWG (minimising excessive GWG). Previous research showed that this type of intervention needed to be person-centred and framed in a sensitive and non-stigmatising format [64–66] for this target group who regularly face stigma within healthcare settings. The integration of PPI in all aspects of intervention development aided this adaptation and, it is hoped, has ultimately improved the quality and appropriateness for the target audience [101].

This habit-based behaviour change intervention has the potential to support women with management of GWG and weight trajectory during the postpartum stage via establishing healthy eating and physical activity behaviours to aid energy balance, with a novel aspect being the focus on the formation of habitual behaviours. The intervention encourages the development of habitual behaviours, by supporting women to enact each specific tip repeatedly in response to a specific context/cue, to facilitate the development of automaticity. This focus on habit-formation makes the HHIPBe intervention different from previous pregnancy-based ‘lifestyle’ interventions. This reproductive period is recognised to involve major life-change for women, where many factors may negatively impact motivation to engage in healthy behaviours, such as sleep deprivation and lack of time [102]. The HHIPBe intervention also maximises the opportunity for behavioural change by embedding key BCTs to support the development of habitual behaviour in the early stages of behavioural enactment, theoretically making it easier for women to enact these behaviours with less effort into the postpartum period. However, a potential limitation to this intervention is the degree to which BCTs can be implemented from self-directed intervention materials (e.g. the leaflet). In addition, the rapid review identified BCTs which were most frequently used in successful GWG interventions, however, these may not necessarily be the most effective BCTs as research on the effectiveness was limited. Engagement with the intervention and the BCTs will be important to assess during the feasibility trial.

### PPI reflections (GRIPP2 SF heading)

It is several decades on since the ‘*nothing about me without me*’ [103] movement gained traction and PPI is now viewed as essential in all research, not only from an ethical and moral stance, but also because of its impact on research quality and potential to increase engagement in research studies [104, 105]. This can be achieved through for example, better acceptability of interventions and trial approaches, better recruitment and retention, providing insights to inform analysis and reporting of trial outcomes and more effective communication and dissemination strategies [101, 105]. For HHIPBe, all PPI collaborators shaped the intervention materials through the lens of women who have personal experience of overweight and/or obesity, and who have recently been pregnant and know the realities of how this feels and the challenges it brings when it comes to engaging in health-promoting diet and physical activity behaviours.

Having PPI collaborators ensured the language and tone used throughout the materials was appropriate, accessible and that the ten tips were realistic for pregnant women. A range of flexible approaches were used to facilitate PPI as they all had young children, some were on maternity leave and some were working outside the home (e.g., emails, text messages, phone calls, posting hard copies of materials which could be annotated with stamped addressed envelopes to return). Initially, recruitment was slow regarding PPI collaborators; both due to limited interest and then compounded by a lack of suitability for the role for those women who did express interest, for example, women who did not have experience of having excess weight, or women who wanted to *participate in the research* study, rather than help to develop the study in partnership. This would suggest that clearer framing of PPI may be needed in future to make it clear how it differs to study participation.

Although ten women initially agreed to collaborate, only six of those ever became actively involved and provided feedback on the study and this may reflect that this is a very busy phase of life for women. From our previous experience of conducting research with women at this stage of life [106], we were cognisant that face-to-face meetings would be challenging due to work or childcare commitments. As a result of employing a flexible and adaptable approach, they engaged in a timely manner and reported that they enjoyed being involved with the development of the intervention. PPI will continue to be involved throughout the length of the study and will be involved during the dissemination of the findings.

The HHIPBe intervention has been developed for testing in a feasibility and acceptability study embedded in routine antenatal care (reported separately); this will provide further insight into the value of this PPI activity by assessing participant views on acceptability and usefulness of the intervention, before proceeding to a definitive RCT.

## Conclusion

The original 10TT was adapted using the Medical Research Council’s guidance on developing complex interventions with PPI and stakeholder input to be suitable for pregnancy. PPI was integral to the development of every aspect of the intervention, providing invaluable feedback and perspectives that shaped the resulting HHIPBe intervention materials. The acceptability of the intervention materials, and the feasibility of embedding a brief habit-based behaviour change intervention within routine antenatal care pathways is now subject to testing in a feasibility randomised controlled trial with women with overweight and obesity (https://clinicaltrials.gov/ct2/show/NCT04336878 ) (reported elsewhere).

## Supplementary Information

Supplementary Material 1.

Supplementary Material 2.

Supplementary Material 3.

## Data Availability

The datasets used and/or analysed during the current study are available from the corresponding author on reasonable request.

## Abbreviations

PPI: personal and public involvement in research (as per Northern Ireland’s definition)
HHIPBe: ‘Healthy Habits in Pregnancy and Beyond’ intervention
GWG: gestational weight gain
10TT: ‘Ten Top Tips for a Healthy Weight’
BCTs: behaviour change techniques
BMI: body mass index
UK: United Kingdom
ROI: Republic of Ireland

## References

[1] Zhao R, Xu L, Wu ML, Huang SH, Cao XJ. Maternal pre-pregnancy body mass index, gestational weight gain influence birth weight. Women Birth J Aust Coll Midwives. 2018;31:20–5 http://www.ncbi.nlm.nih.gov/pubmed/28716548. 10.1016/j.wombi.2017.06.003 28716548

[2] Goldstein RF, Abell SK, Ranasinha S, Misso M, Boyle JA, Black MH, et al. Association of gestational weight gain with maternal and infant outcomes: a systematic review and meta-analysis. JAMA - J Am Med Assoc. 2017;317:2207–25. 10.1001/jama.2017.3635 PMC5815056 28586887

[3] The American College of Obstetricians and Gynecologists (ACOG). ACOG Committee Opinion. 2013 [cited 2018 Dec 11]. https://www.acog.org/Clinical-Guidance-and-Publications/Committee-Opinions/Committee-on-Obstetric-Practice/Weight-Gain-During-Pregnancy?IsMobileSet=false.

[4] Shieh C, Cullen DL, Pike C, Pressler SJ. Intervention strategies for preventing excessive gestational weight gain: systematic review and meta-analysis. Obes Rev. 2018;19:1093–109. 10.1111/obr.12691. 29806187 10.1111/obr.12691

[5] Vincze L, Rollo M, Hutchesson M, Hauck Y, MacDonald-Wicks L, Wood L et al. Interventions including a nutrition component aimed at managing gestational weight gain or postpartum weight retention. JBI Database Syst Rev Implement Reports. 2019 [cited 2019 Mar 19];17:297–364. Available from: http://insights.ovid.com/crossref?an=01938924-201903000-00007. 10.11124/JBISRIR-2017-003593 30870329

[6] Yeo S, Walker JS, Caughey MC, Ferraro AM, Asafu-Adjei JK. What characteristics of nutrition and physical activity interventions are key to effectively reducing weight gain in obese or overweight pregnant women? A systematic review and meta-analysis. Obes Rev. 2017;18:385–99. Available from: http://ovidsp.ovid.com/ovidweb.cgi?T=JS&PAGE=reference&D=med13&NEWS=N&AN=28177566. 10.1111/obr.12511 28177566

[7] Walker R, Bennett C, Blumfield M, Gwini S, Ma J, Wang F et al. Attenuating Pregnancy Weight Gain—What Works and Why: A Systematic Review and Meta-Analysis. Nutrients. 2018 [cited 2019 Feb 25];10:944. Available from: www.mdpi.com/journal/nutrients. 10.3390/nu10070944 PMC6073617 30037126

[8] Agha M, Agha RA, Sandell J. Interventions to Reduce and Prevent Obesity in Pre-Conceptual and Pregnant Women: A Systematic Review and Meta-Analysis. Rosenfeld CS, editor. PLoS One. 2014;9:e95132. Available from: http://ovidsp.ovid.com/ovidweb.cgi?T=JS&PAGE=reference&D=med10&NEWS=N&AN=24827704. 10.1371/journal.pone.0095132 PMC4020754 24827704

[9] Muktabhant B, Lawrie TA, Lumbiganon P, Laopaiboon M. Diet or exercise, or both, for preventing excessive weight gain in pregnancy. Cochrane Database Syst Rev. 2015;CD007145. Available from: http://ovidsp.ovid.com/ovidweb.cgi?T=JS&PAGE=reference&D=medc&NEWS=N&AN=26068707. 10.1002/14651858.CD007145.pub3 PMC9428894 26068707

[10] Campbell F, Johnson M, Messina J, Guillaume L, Goyder E. Behavioural interventions for weight management in pregnancy: A systematic review of quantitative and qualitative data. BMC Public Health. 2011;11:491. Available from: http://ovidsp.ovid.com/ovidweb.cgi?T=JS&PAGE=reference&D=med7&NEWS=N&AN=21696589. 10.1186/1471-2458-11-491 PMC3154865 21696589

[11] Dalrymple KV, Flynn AC, Relph SA, O’Keeffe M, Poston L, Dalrymple KV et al. Lifestyle Interventions in Overweight and Obese Pregnant or Postpartum Women for Postpartum Weight Management: A Systematic Review of the Literature. Nutrients. 2018 [cited 2019 Mar 25];10:1704. Available from: http://www.mdpi.com/2072-6643/10/11/1704. 10.3390/nu10111704 PMC6265993 30405088

[12] Lamminpää R, Vehviläinen-Julkunen K, Schwab U. A systematic review of dietary interventions for gestational weight gain and gestational diabetes in overweight and obese pregnant women. Eur J Nutr. 2018;57:1721–36. Available from: /pmc/articles/PMC6060815/?report = abstract. 29128995 10.1007/s00394-017-1567-z PMC6060815

[13] Campbell F, Messina J, Johnson M, Guillaume L, Madan J, Goyder E et al. Systematic review of dietary and/or physical activity interventions for weight management in pregnancy. 2010. Available from: https://www.nice.org.uk/guidance/ph27/documents/weight-management-in-pregnancy-evidence-review-scharr2.

[14] Vinter CA, Jensen DM, Ovesen P, Beck-Nielsen H, Jørgensen JS. The LiP (Lifestyle in Pregnancy) Study. Diabetes Care. 2011 [cited 2020 Oct 19];34:2502–7. Available from: http://care.diabetesjournals.org/cgi/doi/10.2337/dc11-1150. 10.2337/dc11-1150 PMC3220844 21972411

[15] Wolff S, Legarth J, Vangsgaard K, Toubro S, Astrup A. A randomized trial of the effects of dietary counseling on gestational weight gain and glucose metabolism in obese pregnant women. Int J Obes. 2008;32:495–501. Available from: http://www.nature.com/articles/0803710. 10.1038/sj.ijo.0803710 18227847

[16] Quinlivan JA, Lam LT, Fisher J. A randomised trial of a four-step multidisciplinary approach to the antenatal care of obese pregnant women. Aust New Zeal J Obstet Gynaecol. 2011 [cited 2019 Jul 31];51:141–6. Available from: http://doi.wiley.com/10.1111/j.1479-828X.2010.01268.x. 10.1111/j.1479-828X.2010.01268.x 21466516

[17] Haby K, Berg M, Gyllensten H, Hanas R, Premberg Å. Mighty Mums – a lifestyle intervention at primary care level reduces gestational weight gain in women with obesity. BMC Obes. 2018;5:16. Available from: 10.1186/s40608-018-0194-4. 10.1186/s40608-018-0194-4 PMC5985595 29881627

[18] Phelan S, Wing RR, Brannen A, McHugh A, Hagobian TA, Schaffner A et al. Randomized controlled clinical trial of behavioral lifestyle intervention with partial meal replacement to reduce excessive gestational weight gain. Am J Clin Nutr. 2018 [cited 2019 Jun 24];107:183–94. Available from: https://academic.oup.com/ajcn/article/107/2/183/4911447. 10.1093/ajcn/nqx043 PMC6455030 29529157

[19] NMPA Project Team. National Maternity and Perinatal Audit. London; 2017 [cited 2023 Aug 14]. Available from: https://maternityaudit.org.uk/downloads/RCOGNMPAClinicalReport(web).pdf.

[20] Public Health Agency Health Intelligence Unit. Children’s Health in Northern Ireland. 2017. Available from: https://www.publichealth.hscni.net/sites/default/files/RUAG Childrens Health in NI - 2016-17 - FINAL - Dec 2017.pdf.

[21] Cleo G, Beller E, Glasziou P, Isenring E, Thomas R. Efficacy of habit-based weight loss interventions: a systematic review and meta-analysis. J Behav Med. 2020 [cited 2021 Dec 4];43:519–32. Available from: https://link.springer.com/article/10.1007/s10865-019-00100-w. 10.1007/s10865-019-00100-w 31529279

[22] Kwasnicka D, Dombrowski SU, White M, Sniehotta F. Theoretical explanations for maintenance of behaviour change: a systematic review of behaviour theories. Health Psychol Rev. 2016 [cited 2023 Jun 4];10:277–96. Available from: https://pubmed.ncbi.nlm.nih.gov/26854092/. 10.1080/17437199.2016.1151372 PMC4975085 26854092

[23] Lally P, Gardner B. Promoting habit formation. Health Psychol Rev. 2013;7:S137–58. Available from: http://www.tandfonline.com/doi/abs/10.1080/17437199.2011.603640.

[24] Gardner B, Lally P, Wardle J. Making health habitual: the psychology of ‘habit-formation’ and general practice. Br J Gen Pract. 2012;62:664–6. Available from: https://bjgp.org/lookup/doi/10.3399/bjgp12X659466. 10.3399/bjgp12X659466 PMC3505409 23211256

[25] Gardner B. A review and analysis of the use of ‘habit’ in understanding, predicting and influencing health-related behaviour. Health Psychol Rev. 2015;9:277–95. Available from: https://www.tandfonline.com/doi/full/10.1080/17437199.2013.876238. 10.1080/17437199.2013.876238 PMC4566897 25207647

[26] Beeken RJ, Leurent B, Vickerstaff V, Wilson R, Croker H, Morris S et al. A brief intervention for weight control based on habit-formation theory delivered through primary care: results from a randomised controlled trial. Int J Obes. 2017;41:246–54. Available from: http://www.nature.com/articles/ijo2016206. 10.1038/ijo.2016.206 PMC5300101 27867204

[27] Lally P, Chipperfield A, Wardle J. Healthy habits: efficacy of simple advice on weight control based on a habit-formation model. Int J Obes. 2008 [cited 2018 Dec 12];32:700–7. Available from: http://www.ncbi.nlm.nih.gov/pubmed/18071344. 10.1038/sj.ijo.0803771 18071344

[28] Beeken RJ, Croker H, Morris S, Leurent B, Omar R, Nazareth I et al. Study protocol for the 10 Top Tips (10TT) Trial: Randomised controlled trial of habit-based advice for weight control in general practice. BMC Public Health. 2012;12:667. Available from: https://bmcpublichealth.biomedcentral.com/articles/10.1186/1471-2458-12-667. 10.1186/1471-2458-12-667 PMC3490723 22898059

[29] Patel N, Beeken RJ, Leurent B, Omar RZ, Nazareth I, Morris S. Cost-effectiveness of habit-based advice for weight control versus usual care in general practice in the Ten Top Tips (10TT) trial: economic evaluation based on a randomised controlled trial. BMJ Open. 2018 [cited 2023 Aug 8];8:e017511. Available from: https://bmjopen.bmj.com/content/8/8/e017511. 10.1136/bmjopen-2017-017511 PMC6091904 30104307

[30] Carels RA, Burmeister JM, Koball AM, Oehlhof MW, Hinman N, LeRoy M, et al. A randomized trial comparing two approaches to weight loss: differences in weight loss maintenance. J Health Psychol. 2014;19:296–311. Available from: /pmc/articles/PMC3883879/. 23349402 10.1177/1359105312470156 PMC3883879

[31] McGowan L, Cooke LJ, Gardner B, Beeken RJ, Croker H, Wardle J. Healthy feeding habits: efficacy results from a cluster-randomized, controlled exploratory trial of a novel, habit-based intervention with parents. Am J Clin Nutr. 2013;98:769–77. Available from: https://linkinghub.elsevier.com/retrieve/pii/S0002916523052383. 10.3945/ajcn.112.052159 23864536

[32] Gardner B, Rebar AL, Lally P. A matter of habit: Recognizing the multiple roles of habit in health behaviour. Br J Health Psychol. 2019;24:241–9. Available from: https://onlinelibrary.wiley.com/doi/10.1111/bjhp.12369. 10.1111/bjhp.12369 30945793

[33] Craig P, Dieooe P, Macintyre S, Michie S, Nazareth I, Petticrew M. Developing and evaluating complex interventions. Med Res Counc. 2008;1–39. Available from: https://mrc.ukri.org/documents/pdf/complex-interventions-guidance/. 10.1136/bmj.a1655 PMC2769032 18824488

[34] Hoffmann TC, Glasziou PP, Boutron I, Milne R, Perera R, Moher D et al. Better reporting of interventions: template for intervention description and replication (TIDieR) checklist and guide. BMJ. 2014 [cited 2022 Feb 1];348:g1687–g1687. Available from: https://www.bmj.com/content/348/bmj.g1687. 10.1136/bmj.g1687 24609605

[35] Duncan E, O’Cathain A, Rousseau N, Croot L, Sworn K, Turner KM et al. Guidance for reporting intervention development studies in health research (GUIDED): an evidence-based consensus study. BMJ Open. 2020 [cited 2023 Sep 19];10:e033516. Available from: https://bmjopen.bmj.com/content/10/4/e033516. 10.1136/bmjopen-2019-033516 PMC7245409 32273313

[36] Michie S, Richardson M, Johnston M, Abraham C, Francis J, Hardeman W et al. The Behavior Change Technique Taxonomy (v1) of 93 Hierarchically Clustered Techniques: Building an International Consensus for the Reporting of Behavior Change Interventions. Ann Behav Med. 2013 [cited 2019 Mar 14];46:81–95. Available from: http://openaccess.city.ac.uk/. 10.1007/s12160-013-9486-6 23512568

[37] Soltani H, Arden MA, Duxbury AMS, Fair FJ. An Analysis of Behaviour Change Techniques Used in a Sample of Gestational Weight Management Trials. J Pregnancy. 2016;2016:1–15. Available from: http://www.hindawi.com/journals/jp/2016/1085916/. 10.1155/2016/1085916 PMC4789468 27034836

[38] Kliemann N, Vickerstaff V, Croker H, Johnson F, Nazareth I, Beeken RJ. The role of self-regulatory skills and automaticity on the effectiveness of a brief weight loss habit-based intervention: secondary analysis of the 10 top tips randomised trial. Int J Behav Nutr Phys Act. 2017;14(1):119. Available from: http://ijbnpa.biomedcentral.com/articles/10.1186/s12966-017-0578-8. 10.1186/s12966-017-0578-8 PMC5583960 28870208

[39] Kliemann N. The impact of eating self-regulatory skills on weight control and dietary behaviours in adults. University College London; 2017.

[40] Michie S, Ashford S, Sniehotta FF, Dombrowski SU, Bishop A, French DP. A refined taxonomy of behaviour change techniques to help people change their physical activity and healthy eating behaviours: The CALO-RE taxonomy. Psychol Healt. 2011;26:1479–98. Available from: http://www.tandfonline.com/doi/abs/10.1080/08870446.2010.540664. 10.1080/08870446.2010.540664 21678185

[41] Hartmann-Boyce J, Aveyard P, Koshiaris C, Jebb SA. Development of tools to study personal weight control strategies: OxFAB taxonomy. Obesity. 2016;24:314–20. Available from: http://doi.wiley.com/10.1002/oby.21341. 10.1002/oby.21341 PMC4744943 26748902

[42] Cleo G, Hersch J, Thomas R. Participant experiences of two successful habit-based weight-loss interventions in Australia: a qualitative study. BMJ Open. 2018;8:e020146. Available from:https://bmjopen.bmj.com/lookup/doi/10.1136/bmjopen-2017-020146. 10.1136/bmjopen-2017-020146 PMC5988089 29858412

[43] Hart J, Furber C, Chisholm A, Aspinall S, Lucas C, Runswick E et al. A mixed methods investigation of an online intervention to facilitate student midwives’ engagement in effective conversations about weight-related behaviour change with pregnant women. Midwifery. 2018 [cited 2021 Oct 27];63:52–9. Available from: https://pubmed.ncbi.nlm.nih.gov/29803013/. 10.1016/j.midw.2018.05.001 29803013

[44] Weeks A, Halili L, Ferraro ZM, Harvey AL, Deonandan R, Adamo KB. A Pilot Study Evaluating the Effectiveness of the 5As of Healthy Pregnancy Weight Gain. J Midwifery Womens Health. 2020 [cited 2021 Oct 26];65:546–54. Available from: https://pubmed.ncbi.nlm.nih.gov/32270589/. 10.1111/jmwh.13081 32270589

[45] Canadian Obesity Network. 5A’s of Healthy Pregnancy Weight Gain. 2013. Available from:www.obesitynetwork.ca.

[46] Public Health England. Let ’s Talk About Weight: A step-by-step guide to brief interventions with adults for health and care professionals About Public Health England. Nuf. Dep. Prim. Care- Heal. Sci. 2017. Available from: https://assets.publishing.service.gov.uk/media/5b8d54d2e5274a0bd7d11928/weight_management_toolkit_Let_s_talk_about_weight.pdf.

[47] Health Service Executive. Making Every Contact Count training programme - HSE.ie. [cited 2021 Mar 31]. https://www.hse.ie/eng/about/who/healthwellbeing/making-every-contact-count/training-programme/.

[48] INVOLVE. Briefing notes for researchers:involving the public in NHS, public health and social care research. Eastleigh. 2012. https://www.invo.org.uk/wp-content/uploads/2012/04/INVOLVEBriefingNotesApr2012.pdf.

[49] NIHR. Briefing notes for researchers - public involvement in NHS, health and social care research. 2021 [cited 2021 Jun 3]. https://www.nihr.ac.uk/documents/briefing-notes-for-researchers-public-involvement-in-nhs-health-and-social-care-research/27371.

[50] Staniszewska S, Brett J, Simera I, Seers K, Mockford C, Goodlad S et al. GRIPP2 reporting checklists: tools to improve reporting of patient and public involvement in research. BMJ. 2017 [cited 2020 Apr 29];358:j3453. Available from:https://www.bmj.com/lookup/doi/10.1136/bmj.j3453. 10.1136/bmj.j3453 PMC5539518 28768629

[51] NIHR INVOLVE. INVOLVE guidance on payment and recognition for personal and public involvement in research. 2019 [cited 2020 Oct 29]. https://www.invo.org.uk/resource-centre/payment-and-recognition-for-public-involvement/.

[52] Di Cesare M, Bentham J, Stevens GA, Zhou B, Danaei G, Lu Y et al. Trends in adult body-mass index in 200 countries from 1975 to 2014: a pooled analysis of 1698 population-based measurement studies with 19·2 million participants. Lancet. 2016 [cited 2019 Jan 30];387:1377–96. Available from: www.thelancet.com. 10.1016/S0140-6736(16)30054-X PMC7615134 27115820

[53] Centre for Maternal and Child Enquires. Maternal obesity in the UK: findings from a national project. CMACE. London. 2010. Available from: https://www.publichealth.hscni.net/sites/default/files/Maternal Obesity in the UK.pdf.

[54] Knight M, Bunch K, Tuffnell D, Shakespeare J, Kotnis R, Kenyon S et al. Saving Lives, Improving Mothers’ Care Maternal, Newborn and Infant Clinical Outcome Review Programme. 2020. Available from: www.hqip.org.uk/national-programmes.

[55] Kalliala I, Markozannes G, Gunter MJ, Paraskevaidis E, Gabra H, Mitra A et al. Obesity and gynaecological and obstetric conditions: umbrella review of the literature. BMJ. 2017 [cited 2019 Jan 19];359:j4511. Available from: http://www.ncbi.nlm.nih.gov/pubmed/29074629. 10.1136/bmj.j4511 PMC5656976 29074629

[56] Marchi J, Berg M, Dencker A, Olander EK, Begley C. Risks associated with obesity in pregnancy, for the mother and baby: a systematic review of reviews. Obes Rev. 2015;16:621–38. Available from: https://onlinelibrary.wiley.com/doi/10.1111/obr.12288. 10.1111/obr.12288 26016557

[57] Vats H, Saxena R, Sachdeva MP, Walia GK, Gupta V. Impact of maternal pre-pregnancy body mass index on maternal, fetal and neonatal adverse outcomes in the worldwide populations: A systematic review and meta-analysis. Obes Res Clin Pract. 2021 [cited 2022 Mar 15];15:536–45. Available from: https://pubmed.ncbi.nlm.nih.gov/34782256/. 10.1016/j.orcp.2021.10.005 34782256

[58] D’Souza R, Horyn I, Pavalagantharajah S, Zaffar N, Jacob C-E. Maternal body mass index and pregnancy outcomes: a systematic review and metaanalysis. Am J Obstet Gynecol MFM. 2019 [cited 2022 Mar 15];1:100041. Available from: https://pubmed.ncbi.nlm.nih.gov/33345836/. 10.1016/j.ajogmf.2019.100041 33345836

[59] Poston L, Caleyachetty R, Cnattingius S, Corvalán C, Uauy R, Herring S, et al. Preconceptional and maternal obesity: epidemiology and health consequences. Lancet Diabetes Endocrinol. 2016;4:1025–36 Available from: www.thelancet.com/. 10.1016/S2213-8587(16)30217-0 27743975

[60] Mulherin K, Miller YD, Barlow FK, Diedrichs PC, Thompson R. Weight stigma in maternity care: women’s experiences and care providers’ attitudes. BMC Pregnancy Childbirth. 2013;13:19. Available from: https://bmcpregnancychildbirth.biomedcentral.com/articles/10.1186/1471-2393-13-19. 10.1186/1471-2393-13-19 PMC3577669 23339533

[61] Hill B, Incollingo Rodriguez AC. Weight Stigma across the Preconception, Pregnancy, and Postpartum Periods: A Narrative Review and Conceptual Model. Semin Reprod Med. 2020 [cited 2021 Aug 23];38:414–22. Available from: https://pubmed.ncbi.nlm.nih.gov/33728621/. 10.1055/s-0041-1723775 33728621

[62] Olander EK, Darwin ZJ, Atkinson L, Smith DM, Gardner B. Beyond the ‘teachable moment’ – A conceptual analysis of women’s perinatal behaviour change. Women and Birth. 2016;29:e67–71. Available from: https://linkinghub.elsevier.com/retrieve/pii/S1871519215003510. 10.1016/j.wombi.2015.11.005 26626592

[63] Phelan S. Pregnancy: a teachable moment for weight control and obesity prevention. Am J Obstet Gynecol. 2010;202:135. Available from: https://linkinghub.elsevier.com/retrieve/pii/S0002937809006280. 10.1016/j.ajog.2009.06.008 PMC2815033 19683692

[64] Faucher MA, Mirabito AM. Pregnant Women with Obesity Have Unique Perceptions About Gestational Weight Gain, Exercise, and Support for Behavior Change. J Midwifery Womens Health. 2020;65:529–37. Available from: https://onlinelibrary.wiley.com/doi/10.1111/jmwh.13094. 10.1111/jmwh.13094 32558219

[65] Furber CM, McGowan L. A qualitative study of the experiences of women who are obese and pregnant in the UK. Midwifery. 2011 [cited 2019 Apr 4];27:437–44. Available from: https://www.sciencedirect.com/science/article/pii/S0266613810000562. 10.1016/j.midw.2010.04.001 20483513

[66] Holton S, East C, Fisher J. Weight management during pregnancy: a qualitative study of women’s and care providers’ experiences and perspectives. BMC Pregnancy Childbirth 2017 171. 2017 [cited 2021 Oct 14];17:1–10. Available from:https://bmcpregnancychildbirth.biomedcentral.com/articles/10.1186/s12884-017-1538-7. 10.1186/s12884-017-1538-7 PMC5637069 29020931

[67] Gardner B, Phillips LA, Judah G. Habitual instigation and habitual execution: Definition, measurement, and effects on behaviour frequency. Br J Health Psychol. 2016;21:613–30. Available from: https://onlinelibrary.wiley.com/doi/10.1111/bjhp.12189. 10.1111/bjhp.12189 26991427

[68] Lally P, Wardle J, Gardner B. Experiences of habit formation: a qualitative study. Psychol Heal Med. 2011;16:484–9. 10.1080/13548506.2011.555774 21749245

[69] Gardner B, De Bruijn GJ, Lally P. A systematic review and meta-analysis of applications of the self-report habit index to nutrition and physical activity behaviours. Ann Behav Med. 2011;42:174–87. 21626256 10.1007/s12160-011-9282-0

[70] Neal DT, Wood W, Labrecque JS, Lally P. How do habits guide behavior? Perceived and actual triggers of habits in daily life. J Exp Soc Psychol. 2012;48:492–8. Available from: https://linkinghub.elsevier.com/retrieve/pii/S002210311100254X.

[71] Gardner B, Rebar AL. Habit Formation and Behavior Change. Oxford Res Encycl Psychol. 2019 [cited 2021 Oct 22]. Available from: https://oxfordre.com/psychology/view/10.1093/acrefore/9780190236557.001.0001/acrefore-9780190236557-e-129?_scpsug=crawled%2C3983%2Cen_82cedcf620ef92ed2f9a847c0ce09bf5a1b4529d45732f77150d8f85e05c2164.

[72] Wood W, Neal DT. Healthy through habit: Interventions for initiating & maintaining health behavior change. Behav Sci Policy. 2016 [cited 2019 Jan 21];2:71–83. Available from: https://behavioralpolicy.org/wp-content/uploads/2017/05/BSP_vol1is1_Wood.pdf.

[73] Rothman AJ, Sheeran P, Wood W. Reflective and Automatic Processes in the Initiation and Maintenance of Dietary Change. Ann Behav Med. 2009 [cited 2021 Oct 25];38:4–17. Available from: https://academic.oup.com/abm/article/38/suppl_1/s4/4569644. 10.1007/s12160-009-9118-3 19787308

[74] Hill B, Skouteris H, Fuller-Tyszkiewicz M. Interventions designed to limit gestational weight gain: a systematic review of theory and meta-analysis of intervention components. Obes Rev. 2013;14:435–50. Available from: http://doi.wiley.com/10.1111/obr.12022. 10.1111/obr.12022 23534901

[75] Rhodes A, Smith AD, Chadwick P, Croker H, Llewellyn CH. Exclusively Digital Health Interventions Targeting Diet, Physical Activity, and Weight Gain in Pregnant Women: Systematic Review and Meta-Analysis. JMIR mHealth uHealth. 2020 [cited 2021 May 10];8:e18255. Available from: https://mhealth.jmir.org/2020/7/e18255. 10.2196/18255 PMC7382015 32673251

[76] The Department of Health. Start Active, Stay Active A report on physical activity for health from the four home countries’ Chief Medical Officers. 2011. Available from:www.dh.gov.uk.

[77] Health Service Executive, Department of Health and Children. The National Guidelines on Physical Activity for Ireland. 2009. Available from: https://www.hse.ie/eng/about/who/healthwellbeing/our-priority-programmes/heal/heal-docs/the-national-guidelines-on-physical-activity-for-ireland.pdf.

[78] National Institute for Health and Care Excellence (NICE). Weight management before, during and after pregnancy. 2010 [cited 2018 Nov 23]. Available from: https://www.nice.org.uk/guidance/ph27.

[79] National Institute for Health and Care Excellence (NICE). Maternal and child nutrition Guidance. NICE. 2014 [cited 2019 Feb 26]. Available from: https://www.nice.org.uk/guidance/PH11/chapter/4-Recommendations#diet-in-pregnancy.

[80] UK Chief Medical Officers. Physical Activity in Pregnancy Infographic: Guidance. 2017. pp. 1–7.

[81] NHS. Exercise in pregnancy - NHS. 2017 [cited 2019 Apr 12]. Available from: https://www.nhs.uk/conditions/pregnancy-and-baby/pregnancy-exercise/.

[82] BDA. Food Fact Sheet. 2013 [cited 2019 Mar 13]. pp. 5–6. Available from: www.bda.uk.com/foodfacts.

[83] Royal College of Obstetricians and Gynaecologists. Healthy eating and vitamin supplements in pregnancy. 2014. Available from: www.rcog.org.uk.

[84] British Nutrition Foundation. Hydration and pregnancy - British Nutrition Foundation. 2015 [cited 2019 Feb 25]. Available from: https://www.nutrition.org.uk/healthyliving/nutritionforpregnancy/hydration.html.

[85] Health Service Executive. Healthy Eating for Pregnancy. 2012 [cited 2019 Feb 25]. pp. 1–28. Available from: http://www.cuh.hse.ie/Cork-University-Maternity-Hospital/Publications/Healthy-Eating-For-Pregnancy.pdf.

[86] Irish Nutrition + Dietetic Institute. Healthy Eating during Pregnancy. 2016 [cited 2019 Feb 25]. Available from: www.breastfeeding.ie.

[87] Bingham RJ. Latest Evidence on Alcohol and Pregnancy. Nurs Womens Health. 2015 [cited 2019 Mar 4];19:338–44. Available from: https://www.sciencedirect.com/science/article/pii/S175148511530009X. 10.1111/1751-486X.12219 26264798

[88] The Department of Health. UK Chief Medical Officers’ Alcohol Guidelines Review: Summary of the proposed new guidelines. 2015:1–7.

[89] Royal College of Obstetricians and Gynaecologists. Alcohol and pregnancy: information for you. 2015;2015:1–4. Available from: https://www.rcog.org.uk/globalassets/documents/patients/patient-information-leaflets/pregnancy/pi-alcohol-and-pregnancy.pdf.

[90] O’Keeffe LM, Kearney PM, Greene RA, Kenny LC. Alcohol use during pregnancy. Obstet Gynaecol Reprod Med. 2016 [cited 2019 Mar 4];26:188–9. Available from: https://www.sciencedirect.com/science/article/pii/S1751721416300951.

[91] Dugas M, Gao G, Agarwal R. Unpacking mHealth interventions: A systematic review of behavior change techniques used in randomized controlled trials assessing mHealth effectiveness. Digit. Heal. SAGE Publications Inc.; 2020 [cited 2020 Sep 8]. Available from: /pmc/articles/PMC7036494/?report = abstract. 10.1177/2055207620905411 PMC7036494 32128233

[92] Hughson JP, Daly JO, Woodward-Kron R, Hajek J, Story D. The Rise of Pregnancy Apps and the Implications for Culturally and Linguistically Diverse Women: Narrative Review. JMIR mHealth uHealth. 2018 [cited 2023 Aug 13];6:e189. Available from: /pmc/articles/PMC6269626/. 10.2196/mhealth.9119 PMC6269626 30446483

[93] Tripp N, Hainey K, Liu A, Poulton A, Peek M, Kim J et al. An emerging model of maternity care: Smartphone, midwife, doctor? Women and Birth. 2014 [cited 2023 Aug 13];27:64–7. Available from: https://pubmed.ncbi.nlm.nih.gov/24295598/. 10.1016/j.wombi.2013.11.001 24295598

[94] Kliemann N, Croker H, Johnson F, Beeken RJ. Development of the Top Tips Habit-Based Weight Loss App and Preliminary Indications of Its Usage, Effectiveness, and Acceptability: Mixed-Methods Pilot Study. JMIR mHealth uHealth. 2019 [cited 2020 Oct 30];7:e12326. Available from: http://www.ncbi.nlm.nih.gov/pubmed/31094352. 10.2196/12326 PMC6533874 31094352

[95] Schmied VA, Duff M, Dahlen HG, Mills AE, Kolt GS. ‘Not waving but drowning’: a study of the experiences and concerns of midwives and other health professionals caring for obese childbearing women. Midwifery. 2011 [cited 2021 Oct 6];27:424–30. Available from: https://pubmed.ncbi.nlm.nih.gov/20381222/. 10.1016/j.midw.2010.02.010 20381222

[96] Heslehurst N, Lang R, Rankin J, Wilkinson JR, Summerbell CD. Obesity in pregnancy: a study of the impact of maternal obesity on NHS maternity services. BJOG. 2007 [cited 2023 Jun 4];114:334–42. Available from: https://pubmed.ncbi.nlm.nih.gov/17261124/. 10.1111/j.1471-0528.2006.01230.x 17261124

[97] Cunningham J, Endacott R, Gibbons D. Communication with health professionals: The views of pregnant women with a raised BMI. Br. J. Midwifery. 2018 [cited 2023 Jun 4]. Available from: https://www.britishjournalofmidwifery.com/content/research/communication-with-health-professionals-the-views-of-pregnant-women-with-a-raised-bmi/.

[98] Weeks A, Halili L, Ferraro ZM, Harvey ALJ, Deonandan R, Adamo KB. A Pilot Study Evaluating the Effectiveness of the 5As of Healthy Pregnancy Weight Gain. J Midwifery Womens Health. 2020 [cited 2022 May 17];65:546–54. Available from: https://onlinelibrary.wiley.com/doi/full/10.1111/jmwh.13081. 10.1111/jmwh.13081 32270589

[99] Obesity Canada. 5As of Obesity Management. 2011. Available from: https://obesitycanada.ca/5as-adult/.

[100] NI Centre for Pharmacy Learning and Development. NICPLD: Open learning. [cited 2023 Aug 13]. Available from: https://www.nicpld.org/courses/?programme=pharmacist&coursetype=ol&show=allusers.

[101] Brett J, Staniszewska S, Mockford C, Herron-Marx S, Hughes J, Tysall C et al. Mapping the impact of patient and public involvement on health and social care research: a systematic review. Heal Expect. 2014 [cited 2023 Jun 4];17:637–50. Available from: https://pubmed.ncbi.nlm.nih.gov/22809132/. 10.1111/j.1369-7625.2012.00795.x PMC5060910 22809132

[102] McKinley MC, Allen-Walker V, McGirr C, Rooney C, Woodside JV. Weight loss after pregnancy: challenges and opportunities. Nutr Res Rev. 2018;31:225–38. Available from: 10.1017/S0954422418000070. 10.1017/S0954422418000070 29984681

[103] Delbanco T, Berwick DM, Boufford JI, Edgman-Levitan PA, Ollenschläger G, Plamping D et al. Healthcare in a land called PeoplePower: nothing about me without me. Health Expect. 2001 [cited 2023 Jun 4];4:144. Available from: /pmc/articles/PMC5060064. 10.1046/j.1369-6513.2001.00145.x PMC5060064 11493320

[104] Richards T, Snow R, Schroter S. Co-creating health: more than a dream. BMJ. 2016;354:i4550 https://pubmed.ncbi.nlm.nih.gov/27633967/. 10.1136/bmj.i4550 27633967

[105] Russell J, Fudge N, Greenhalgh T. The impact of public involvement in health research: what are we measuring? Why are we measuring it? Should we stop measuring it? Res Involv Engagem. 2020;6:1–8. Available from: https://researchinvolvement.biomedcentral.com/articles/10.1186/s40900-020-00239-w. 10.1186/s40900-020-00239-w PMC7592364 33133636

[106] McGirr C, Rooney C, Gallagher D, Dombrowski SU, Anderson AS, Cardwell CR, et al. Text messaging to help women with overweight or obesity lose weight after childbirth: the intervention adaptation and SMS feasibility RCT. Public Heal Res. 2020;8:1–152. Available from: https://www.journalslibrary.nihr.ac.uk/phr/phr08040. 32223118

