# Supplementary material for: Development of a habit-based intervention to support healthy eating and physical activity behaviours for pregnant women with overweight or obesity: Healthy Habits in Pregnancy and Beyond (HHIPBe)
Source: BMC Pregnancy Childbirth. 2024 Nov 16;24:760. doi: 10.1186/s12884-024-06945-7 (PMC11568677; doi:10.1186/s12884-024-06945-7)
Supplement: Supplementary file 1 — Supplementary Material 1. [file 12884_2024_6945_MOESM1_ESM.docx]

| Item Description | Page in manuscript where item is located | Other* |
| --- | --- | --- |
| 1. Report the context for which the intervention was developed. | Page 6-7 |  |
| 1. Report the purpose of the intervention development process. | Page 7 |  |
| 1. Report the target population for the intervention development | Page 6-7 |  |
| 1. Report how any published intervention development approach contributed to the development process | Page 8 |  |
| 1. Report how evidence from difference sources informed the intervention development process | Page 13-15 |  |
| 1. Report how/if published theory informed the intervention development process | Page 8, 15-18 |  |
| 1. Report any use of components from an existing intervention in the current intervention development process | Page 10-11 |  |
| 1. Report any guiding principles, people or factors that were prioritised when making decisions during the intervention development process. | Page 34 |  |
| 1. Report how stakeholders contributed to the intervention development process. | Page 10-13 |  |
| 1. Report how the intervention changed in content and format from the start of the intervention development process | Page 18-32 |  |
| 1. Report any changes to interventions required or likely to be required for subgroups. | Page 18-32 |  |
| 1. Report important uncertainties at the end of the intervention development process. | Page 36 |  |
| 1. Follow TIDieR guidance when describing the developed intervention. | * | The TIDieR framework was used to describe the intervention. |
| 1. Report the intervention development process in an open access format. | * | This paper will be published in an open access format. |

**Addition File 1: Guided- a guideline for reporting for intervention development studies (Duncan *et.al*, 2020).**
