# Supplementary material for: Development of a habit-based intervention to support healthy eating and physical activity behaviours for pregnant women with overweight or obesity: Healthy Habits in Pregnancy and Beyond (HHIPBe)
Source: BMC Pregnancy Childbirth. 2024 Nov 16;24:760. doi: 10.1186/s12884-024-06945-7 (PMC11568677; doi:10.1186/s12884-024-06945-7)
Supplement: Supplementary file 2 — Supplementary Material 2. [file 12884_2024_6945_MOESM2_ESM.docx]

**Additional File 2 - Search Strategy for Gestational Weight Gain (GWG) interventions**

| **#** | **Query** |
| --- | --- |
| 1 | Pregnancy/or pregnan*.mp. |
| 2 | GWG.mp. or GWG/ |
| 3 | Obesity Management/or Obesity/or Obesity, Maternal/ |
| 4 | Overweight/ |
| 5 | (weight adj3 (control* or management*)).mp. [mp=title, abstract, original title, name of substance word, subject heading word, floating sub-heading word, keyword heading word, organism supplementary concept word, protocol supplementary concept word, rare disease supplementary concept word, unique identifier, synonyms] |
| 6 | (weight adj3 (gain* or change*)).mp. [mp=title, abstract, original title, name of substance word, subject heading word, floating sub-heading word, keyword heading word, organism supplementary concept word, protocol supplementary concept word, rare disease supplementary concept word, unique identifier, synonyms] |
| 7 | obese.mp. or Obesity/or Body Mass Index/ |
| 8 | intervention.mp. |
| 9 | exp health promotion/ |
| 10 | Health Education/ |
| 11 | (health* adj3 (promot* or educat* or lifestyle*)).mp. [mp=title, abstract, original title, name of substance word, subject heading word, floating sub-heading word, keyword heading word, organism supplementary concept word, protocol supplementary concept word, rare disease supplementary concept word, unique identifier, synonyms] |
| 12 | behavio?r therapy.mp. |
| 13 | behavio*r change.mp. |
| 14 | diet.mp. or Diet, Healthy/or Diet/ |
| 15 | nutrition.mp. |
| 16 | 8 or 9 or 10 or 11 or 12 or 13 or 14 or 15 |
| 17 | 1 or 2 |
| 18 | 3 or 4 or 5 or 6 or 7 |
| 19 | 16 and 17 and 18 |
| 20 | limit 19 to (English language and humans) |
| 21 | Limit 20 to systematic review |
