# Supplementary figures and images for: Development of a habit-based intervention to support healthy eating and physical activity behaviours for pregnant women with overweight or obesity: Healthy Habits in Pregnancy and Beyond (HHIPBe)
Source: BMC Pregnancy Childbirth. 2024 Nov 16;24:760. doi: 10.1186/s12884-024-06945-7 (PMC11568677; doi:10.1186/s12884-024-06945-7)

**Additional File 3 – Personal and Public Involvement (PPI) Collaborator Recruitment Posters**


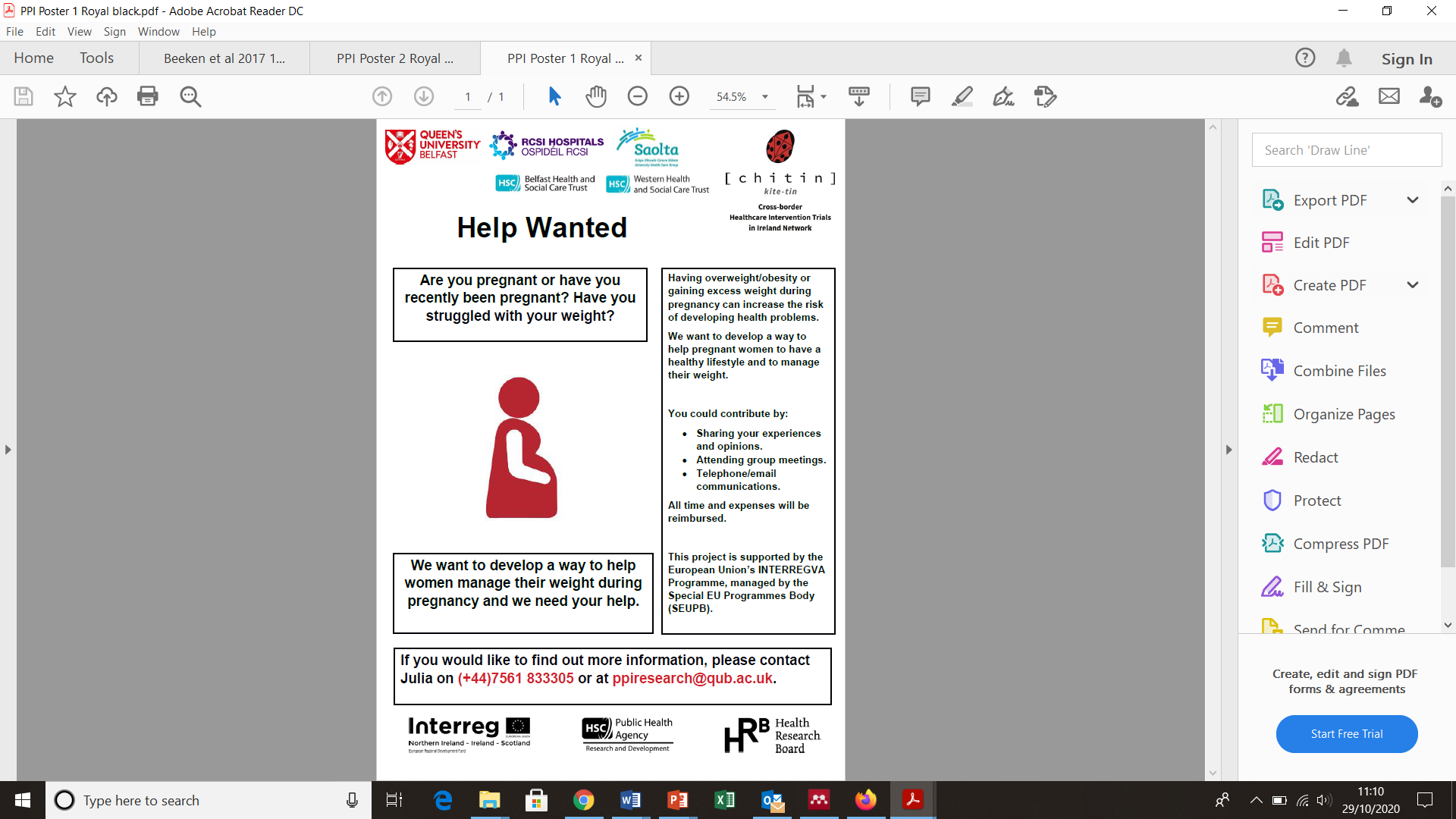


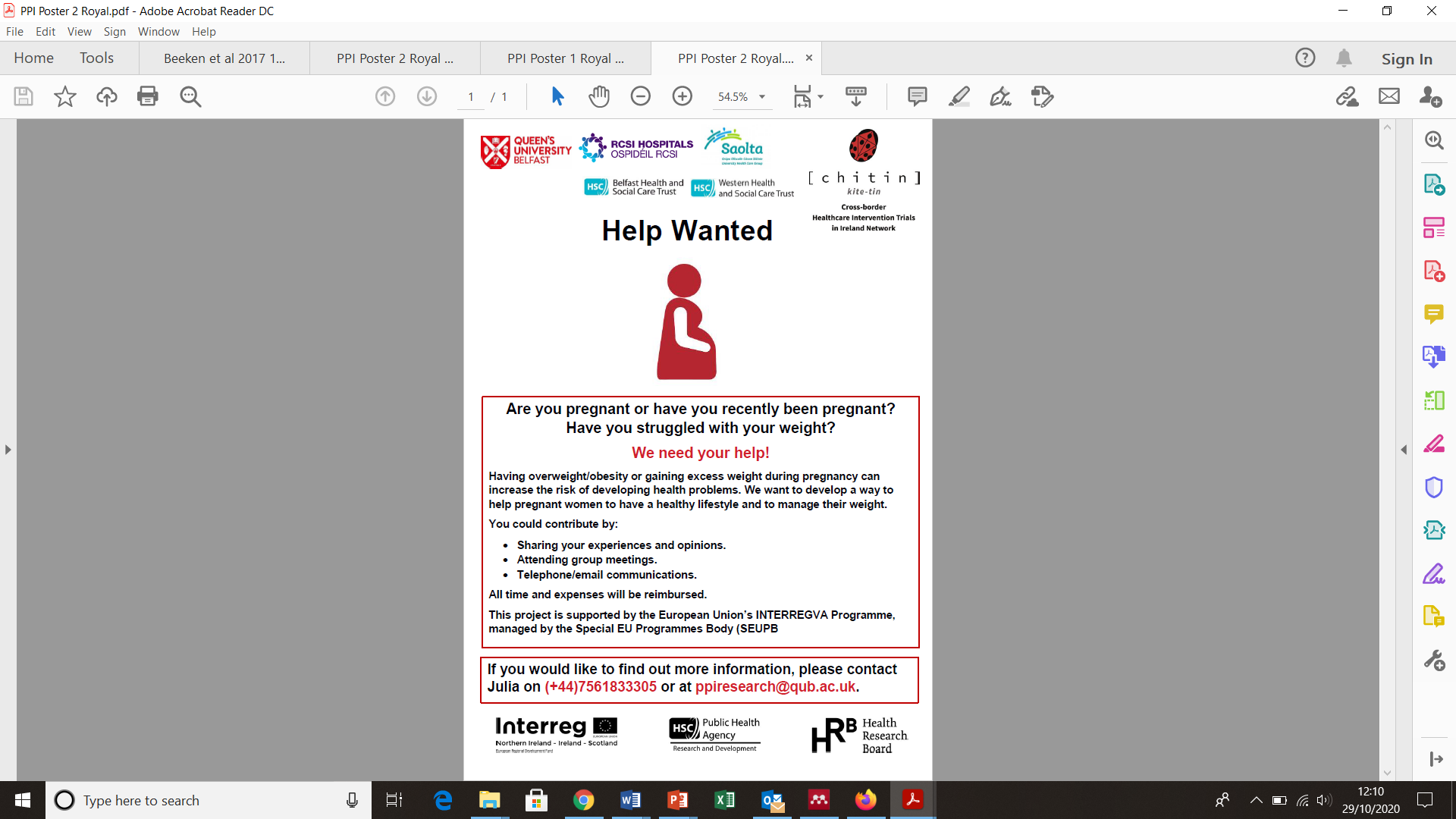

Supplement: Supplementary file 3 — Supplementary Material 3. [file 12884_2024_6945_MOESM3_ESM.docx]
